# Probabilistic Inference in General Graphical Models through Sampling in Stochastic Networks of Spiking Neurons

**DOI:** 10.1371/journal.pcbi.1002294

**Published:** 2011-12-15

**Authors:** Dejan Pecevski, Lars Buesing, Wolfgang Maass

**Affiliations:** Institute for Theoretical Computer Science, Graz University of Technology, Graz, Austria; Indiana University, United States of America

## Abstract

An important open problem of computational neuroscience is the generic organization of computations in networks of neurons in the brain. We show here through rigorous theoretical analysis that inherent stochastic features of spiking neurons, in combination with simple nonlinear computational operations in specific network motifs and dendritic arbors, enable networks of spiking neurons to carry out probabilistic inference through sampling in general graphical models. In particular, it enables them to carry out probabilistic inference in Bayesian networks with converging arrows (“explaining away”) and with undirected loops, that occur in many real-world tasks. Ubiquitous stochastic features of networks of spiking neurons, such as trial-to-trial variability and spontaneous activity, are necessary ingredients of the underlying computational organization. We demonstrate through computer simulations that this approach can be scaled up to neural emulations of probabilistic inference in fairly large graphical models, yielding some of the most complex computations that have been carried out so far in networks of spiking neurons.

## Author Summary

Experimental data from neuroscience have provided substantial knowledge about the intricate structure of cortical microcircuits, but their functional role, i.e. the computational calculus that they employ in order to interpret ambiguous stimuli, produce predictions, and derive movement plans has remained largely unknown. Earlier assumptions that these circuits implement a logic-like calculus have run into problems, because logical inference has turned out to be inadequate to solve inference problems in the real world which often exhibits substantial degrees of uncertainty. In this article we propose an alternative theoretical framework for examining the functional role of precisely structured motifs of cortical microcircuits and dendritic computations in complex neurons, based on probabilistic inference through sampling. We show that these structural details endow cortical columns and areas with the capability to represent complex knowledge about their environment in the form of higher order dependencies among salient variables. We show that it also enables them to use this knowledge for probabilistic inference that is capable to deal with uncertainty in stored knowledge and current observations. We demonstrate in computer simulations that the precisely structured neuronal microcircuits enable networks of spiking neurons to solve through their inherent stochastic dynamics a variety of complex probabilistic inference tasks.

## Introduction

We show in this article that noisy networks of spiking neurons are in principle able to carry out a quite demanding class of computations: probabilistic inference in general graphical models. More precisely, they are able to carry out probabilistic inference for arbitrary probability distributions over discrete random variables (RVs) through sampling. Spikes are viewed here as signals which inform other neurons that a certain RV has been assigned a particular value for a certain time period during the sampling process. This approach had been introduced under the name “neural sampling” in [1]. This article extends the results of [1], where the validity of this neural sampling process had been established for the special case of distributions 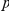 with at most 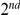 order dependencies between RVs, to distributions 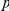 with dependencies of arbitrary order. Such higher order dependencies, which may cause for example the explaining away effect [2], have been shown to arise in various computational tasks related to perception and reasoning. Our approach provides an alternative to other proposed neural emulations of probabilistic inference in graphical models, that rely on arithmetical methods such as belief propagation. The two approaches make completely different demands on the underlying neural circuits: the belief propagation approach emulates a deterministic arithmetical computation of probabilities, and is therefore optimally supported by noise-free deterministic networks of neurons. In contrast, our sampling based approach shows how an internal model of an arbitrary target distribution 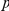 can be implemented by a network of stochastically firing neurons (such internal model for a distribution 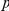, that reflects the statistics of natural stimuli, has been found to emerge in primary visual cortex [3]). This approach requires the presence of stochasticity (noise), and is inherently compatible with experimentally found phenomena such as the ubiquitous trial-to-trial variability of responses of biological networks of neurons.

Given a network of spiking neurons that implements an internal model for a distribution 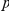, probabilistic inference for 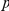, for example the computation of marginal probabilities for specific RVs, can be reduced to counting the number of spikes of specific neurons for a behaviorally relevant time span of a few hundred ms, similarly as in previously proposed mechanisms for evidence accumulation in neural systems [4]. Nevertheless, in this neural emulation of probabilistic inference through sampling, every single spike conveys information, as well as the relative timing among spikes of different neurons. The reason is that for many of the neurons in the model (the so-called principal neurons) each spike represents a tentative value for a specific RV, whose consistency with tentative values of other RVs, and with the available evidence (e.g., an external stimulus), is explored during the sampling process. In contrast, currently known neural emulations of belief propagation in general graphical models are based on firing rate coding.

The underlying mathematical theory of our proposed new method provides a rigorous proof that the spiking activity in a network of neurons can in principle provide an internal model for an arbitrary distribution 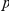. It builds on the general theory of Markov chains and their stationary distribution (see e.g. [5]), the general theory of MCMC (Markov chain Monte Carlo) sampling (see e.g. [6], [7]), and the theory of sampling in stochastic networks of spiking neurons - modelled by a non-reversible Markov chain [1]. It requires further theoretical analysis for elucidating under what conditions higher order factors of p can be emulated in networks of spiking neurons, which is provided in the Methods section of this article. Whereas the underlying mathematical theory only guarantees convergence of the spiking activity to the target distribution 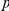, it does not provide tight bounds for the convergence speed to 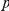 (the so-called burn–in time in MCMC sampling). Hence we complement our theoretical analysis by computer simulations for three Bayesian networks of increasing size and complexity. We also address in these simulations the question to what extent the speed or precision of the probabilistic inference degrades when one moves from a spiking neuron model that is optimal from the perspective of the underlying theory to a biologically more realistic neuron model. The results show, that in all cases quite good probabilistic inference results can be achieved within a time span of a few hundreds ms. In the remainder of this section we sketch the conceptual and scientific background for our approach. An additional discussion of related work can be found in the discussion section.

Probabilistic inference in Bayesian networks [2] and other graphical models [8], [9] is an abstract description of a large class of computational tasks, that subsumes in particular many types of computational tasks that the brain has to solve: The formation of coherent interpretations of incomplete and ambiguous sensory stimuli, integration of previously acquired knowledge with new information, movement planning, reasoning and decision making in the presence of uncertainty [10]–[13]. The computational tasks become special cases of probabilistic inference if one assumes that the previously acquired knowledge (facts, rules, constraints, successful responses) is encoded in a joint distribution 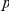 over numerous RVs 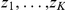, that represent features of sensory stimuli, aspects of internal models for the environment, environmental and behavioral context, values of carrying out particular actions in particular situations [14], goals, etc. If the values of some of these RVs assume concrete values 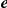 (e.g. because of observations, or because a particular goal has been set), the distribution of the remaining variables changes in general (to the conditional distribution given the values 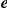). A typical computation that needs to be carried out for probabilistic inference for some joint distribution 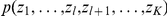 involves in addition marginalization, and requires for example the evaluation of an expression of the form
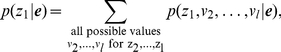
(1)where concrete values 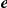 (the “evidence”or “observations” have been inserted for the RVs 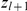, 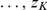. These variables are then often called observable variables, and the others latent variables. Note that the term “evidence” is somewhat misleading, since the assignment 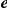 represents some arbitrary input to a probabilistic inference computation, without any connotation that it represents correct observations or memories. The computation of the resulting marginal distribution 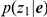 requires a summation over all possible values 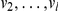 for the RVs 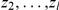 that are currently not of interest for this probabilistic inference. This computation is in general quite complex (in fact, it is NP-complete [9]) because in the worst case exponentially in 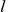 many terms need to be evaluated and summed up.

There exist two completely different approaches for solving probabilistic inference tasks of type (1), to which we will refer in the following as the arithmetical and the sampling approach. In the arithmetical approach one exploits particular features of a graphical model, that captures conditional independence properties of the distribution 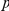, for organizing the order of summation steps and multiplication steps for the arithmetical calculation of the r.h.s. of (1) in an efficient manner. Belief propagation and message passing algorithms are special cases of this arithmetical approach. All previously proposed neural emulations of probabilistic inference in general graphical models have pursued this arithmetical approach. In the sampling approach, which we pursue in this article, one constructs a method for drawing samples from the distribution 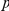 (with fixed values 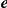 for some of the RVs, see (1)). One can then approximate the l.h.s. of (1), i.e., the desired value of the probability 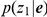, by counting how often each possible value for the RV 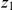 occurs among the samples. More precisely, we identify conditions under which each current firing state (which records which neuron has fired within some time window) of a network of stochastically firing neurons can be viewed as a sample from a probability distribution that converges to the target distribution 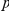. For this purpose the temporal dynamics of the network is interpreted as a (non-reversible) Markov chain. We show that a suitable network architecture and parameter choice of the network of spiking neurons can make sure that this Markov chain has the target distribution 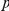 as its stationary distribution, and therefore produces after some “burn–in time”samples (i.e., firing states) from a distribution that converges to 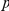. This general strategy for sampling is commonly referred to as Markov chain Monte Carlo (MCMC) sampling [6], [7], [9].

Before the first use of this strategy in networks of spiking neurons in [1], MCMC sampling had already been studied in the context of artificial neural networks, so-called Boltzmann machines [15]. A Boltzmann machine consists of stochastic binary neurons in discrete time, where the output of each neuron has the value 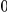 or 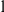 at each discrete time step. The probability of each value depends on the output values of neurons at the preceding discrete time step. For a Boltzmann machine a standard way of sampling is Gibbs sampling. The Markov chain that describes Gibbs sampling is reversible, i.e., stochastic transitions between states do not have a preferred direction in time. This sampling method works well in artificial neural networks, where the effect of each neural activity lasts for exactly one discrete time step. But it is in conflict with basic features of networks of spiking neurons, where each action potential (spike) of a neuron triggers inherent temporal processes in the neuron itself (e.g. refractory processes), and postsynaptic potentials of specific durations in other neurons to which it is synaptically connected. These inherent temporal processes of specific durations are non-reversible, and are therefore inconsistent with the mathematical model (Gibbs sampling) that underlies probabilistic inference in Boltzmann machines. [1] proposed a somewhat different mathematical model (sampling in non-reversible Markov chains) as an alternative framework for sampling, that is compatible with these basic features of the dynamics of networks of spiking neurons.

We consider in this article two types of models for spiking neurons (see Methods for details):

- stochastic leaky integrate –and –fire neurons with absolute and relative refractory periods, formalized in the spike–response framework of [16] (as in [1]), and
- simplified stochastic multi–ompartment neuron models with dendritic spikes.

A key step for interpreting the firing activity of networks of neurons as sampling from a probability distribution (as proposed in [3]) in a rigorous manner is to define a formal relationship between spikes and samples. As in [1] we relate the firing activity in a network 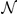 of 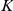 spiking neurons 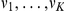 to sampling from a distribution 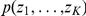 over binary variables 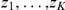 by setting
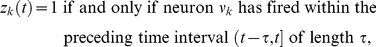
(2)(we restrict our attention here to binary RVs; multinomial RVs could in principle be represented by WTA circuits –see Discussion). The constant 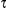 models the average length of the effect of a spike on the firing probability of other neurons or of the same neuron, and can be set for example to 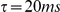.

However with this definition of its internal state (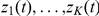) the dynamics of the neural network 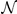 can not be modelled by a Markov chain, since knowledge of this current state does not suffice for determining the distribution of states at future time points, say at time 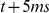. This distribution requires knowledge about when exactly a neuron 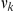 with 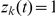 had fired. Therefore auxiliary RVs 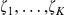 with multinomial or analog values were introduced in [1], that keep track of when exactly in the preceding time interval of length 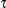 a neuron 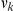 had fired, and thereby restore the Markov property for a Markov chain that is defined over an enlarged state set consisting of all possible values of 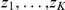 and 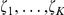. However the introduction of these hidden variables 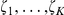, that keep track of inherent temporal processes in the network 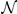 of spiking neurons, comes at the price that the resulting Markov chain is no longer reversible (because these temporal processes are not reversible). But it was shown in [1] that one can prove nevertheless for any distribution 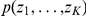 for which the so-called neural computability condition (NCC), see below, can be satisfied by a network 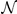 of spiking neurons, that 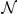 defines a non-reversible Markov chain whose stationary distribution is an expanded distribution 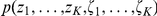, whose marginal distribution over 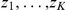 (which results when one ignores the values of the hidden variables 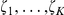) is the desired distribution 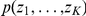. Hence a network 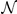 of spiking neurons can sample from any distribution 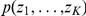 for which the NCC can be satisfied. This implies that any neural system that contains such network 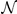 can carry out the probabilistic inference task (1): The evidence 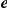 could be implemented through external inputs that force neuron 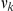 to fire at a high rate if 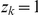 in 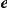, and not to fire if 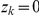 in 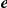. In order to estimate 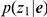, it suffices that some readout neuron estimates (after some initial transient phase) the resulting firing rate of the neuron 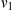 that represents RV 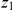.

In contrast to most of the other neural implementations of probabilistic inference (with some exceptions, see for example [17] and [18]) where information is encoded in the firing rate of the neurons, in this approach the spike times, rather than the firing rate, of the neuron 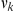 carry relevant information as they define the value of the RV 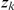 at a particular moment in time 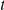 according to (2). In this spike-time based coding scheme, the relative timing of spikes (which neuron fires simultaneously with whom) receives a direct functional interpretation since it determines the correlation between the corresponding RVs.

The NCC requires that for each RV 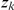 the firing probability density 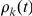 of its corresponding neuron 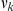 at time 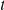 satisfies, if the neuron is not in a refractory period,
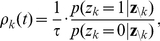
(3)where 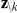 denotes the current value of all other RVs, i.e., all 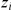 with 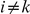. We use in this article the same model for a stochastic neuron as in [1] (continuous time case), which can be matched quite well to biological data according to [19]. In the simpler version of this neuron model one assumes that it has an absolute refractory period of length 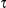, and that the instantaneous firing probability 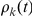 satisfies outside of its refractory period 
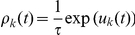
, where 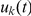 is its membrane potential (see Methods for an account of the more complex neuron model with a relative refractory period from [1], that we have also tested in our simulations). The NCC from (3) can then be reformulated as a condition on the membrane potential of the neuron
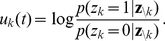
(4)


Let us consider a Boltzmann distribution 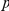 of the form
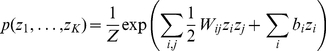
(5)with symmetric weights (i.e., 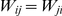) that vanish on the diagonal (i.e., 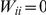). In this case the NCC can be satisfied by a 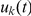 that is *linear* in the postsynaptic potentials that neuron 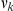 receives from the neurons 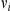 that represent other RVs 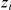:
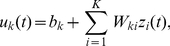
(6)where 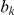 is the bias of neuron 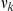 (which regulates its excitability), 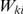 is the strength of the synaptic connection from neuron 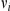 to 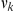, and 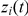 approximates the time course of the postsynaptic potential caused by a firing of neuron 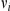 at some time 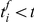 (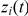 assumes value 1 during the time interval 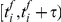, otherwise it has value 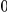).

**Figure 1:**
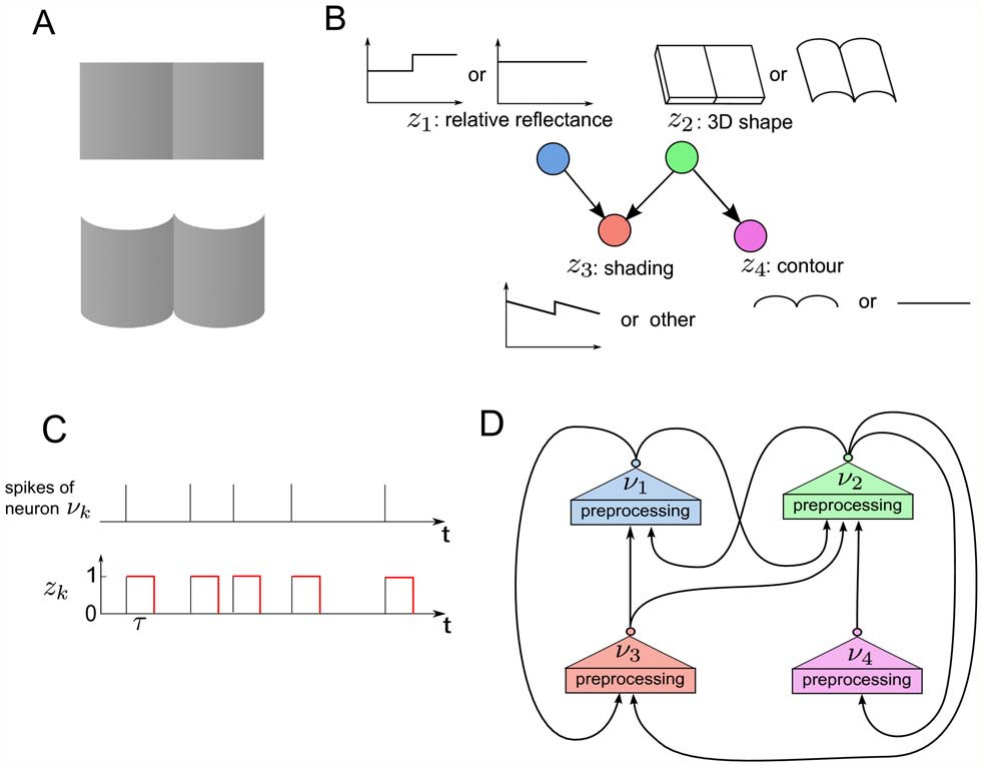
The visual perception experiment of [**21**] that demonstrates “explaining away” and its corresponding Bayesian network model. **A**) Two visual stimuli, each exhibiting the same luminance profile in the horizontal direction, differ only with regard to their contours, which suggest different 3D shapes (flat versus cylindrical). This in turn influences our perception of the reflectance of the two halves of each stimulus (a step in the reflectance at the middle line, versus uniform reflectance): the cylindrical 3D shape “explains away”the reflectance step. **B**) The Bayesian network that models this effect represents the probability distribution 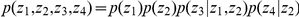. The relative reflectance (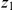) of the two halves is either different (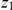 = 1) or the same (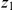 = 0). The perceived 3D shape can be cylindrical (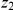 = 1) or flat (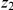 = 0). The relative reflectance and the 3D shape are direct causes of the shading (luminance change) of the surfaces (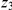), which can have the profile like in panel A (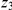 = 1) or a different one (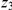 = 0). The 3D shape of the surfaces causes different perceived contours, flat (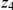 = 0) or cylindrical (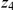 = 1). The observed variables (evidence) are the contour (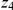) and the shading (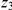). Subjects infer the marginal posterior probability distributions of the relative reflectance 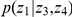 and the 3D shape 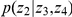 based on the evidence. **C**) The RVs 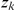 are represented in our neural implementations by principal neurons 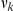. Each spike of 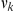 sets the RV 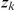 to 1 for a time period of length 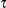. **D**) The structure of a network of spiking neurons that performs probabilistic inference for the Bayesian network of panel B through sampling from conditionals of the underlying distribution. Each principal neuron employs preprocessing to satisfy the NCC, either by dendritic processing or by a preprocessing circuit.

However, it is well known that probabilistic inference for distributions of the form (5) is too weak to model various important computational tasks that the brain is obviously able to solve, at least without auxiliary variables. While (5) only allows pairwise interactions between RVs, numerous real world probabilistic inference tasks require inference for distributions with higher order terms. For example, it has been shown that human visual perception involves “explaining away”, a well known effect in probabilistic inference, where a change in the probability of one competing hypothesis for explaining some observation affects the probability of another competing hypothesis [20]. Such effects can usually only be captured with terms of order at least 3, since 3 RVs (for 2 hypotheses and 1 observation) may interact in complex ways. A well known example from visual perception is shown in Fig. 1, for a probability distribution 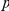 over 4 RVs 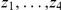, where 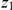 is defined by the perceived relative reflectance of two abutting 2D areas, 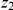 by the perceived 3D shape of the observed object, 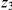 by the observed shading of the object, and 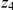 by the contour of the 2D image. The difference in shading of the two abutting surfaces in Fig. 1A could be explained either by a difference in reflectance of the two surfaces, or by an underlying curved 3D shape. The two different contours (RV 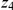) in the upper and lower part of Fig. 1A influence the likelihood of a curved 3D shape (RV 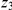). In particular, a perceived curved 3D shape “explains away” the difference in shading, thereby making a uniform reflectance more likely. The results of [21] and numerous related results suggest that the brain is able to carry out probabilistic inference for more complex distributions than the 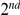 order Boltzmann distribution (5).

We show in this article that the neural sampling method of [1] can be extended to any probability distribution 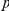 over binary RVs, in particular to distributions with higher order dependencies among RVs, by using auxiliary spiking neurons in 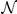 that do not directly represent RVs 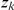, or by using nonlinear computational processes in multi-compartment neuron models. As one can expect, the number of required auxiliary neurons or dendritic branches increases with the complexity of the probability distribution 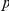 for which the resulting network of spiking neurons has to carry out probabilistic inference. Various types of graphical models [9] have emerged as convenient frameworks for characterizing the complexity of distributions 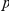 from the perspective of probabilistic inference for 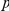.

We will focus in this article on Bayesian networks, a common type of graphical model for probability distributions. But our results can also be applied for other types of graphical models. A Bayesian network is a directed graph (without directed cycles), whose nodes represent RVs 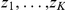. Its graph structure indicates that 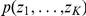 admits a factorization of the form
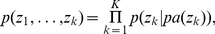
(7)where 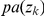 is the set of all (direct) parents of the node indexed by 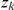. For example, the Bayesian network in Fig. 1B implies that the factorization 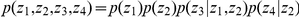 is possible.

We show that the complexity of the resulting network of spiking neurons for carrying out probabilistic inference for 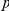 can be bounded in terms of the graph complexity of the Bayesian network that gives rise to the factorization (7). More precisely, we present three different approaches for constructing such networks of spiking neurons:

- through a reduction of 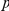 to a Boltzmann distribution (5) with auxiliary RVs
- through a Markov blanket expansion of the r.h.s. of the NCC (4)
- through a factorized expansion of the r.h.s. of the NCC (4)

We will show that there exist two different neural implementation options for each of the last two approaches, using either specific network motifs or dendritic processing for nonlinear computation steps. This yields altogether 5 different options for emulating probabilistic inference in Bayesian networks through sampling via the inherent stochastic dynamics of networks of spiking neurons. We will exhibit characteristic differences in the complexity and performance of the resulting networks, and relate these to the complexity of the underlying Bayesian network. All 5 of these neural implementation options can readily be applied to Bayesian networks where several arcs converge to a node (giving rise to the “explaining away” effect), and to Bayesian networks with undirected cycles (“loops”). All methods for probabilistic inference from general graphical models that we propose in this article are from the mathematical perspective special cases of MCMC sampling. However in view of the fact that they expand the neural sampling approach of [1], we will refer to them more specifically as neural sampling.

We show through computer simulations for three different Bayesian networks of different sizes and complexities that neural sampling can be carried quite fast with the help of the second and third approach, providing good inference results within a behaviorally relevant time span of a few hundred ms. One of these Bayesian networks addresses the previously described classical “explaining away” effect in visual perception from Fig. 1. The other two Bayesian networks not only contain numerous “explaining away” effects, but also undirected cycles. Altogether, our computer simulations and our theoretical analyses demonstrate that networks of spiking neurons can emulate probabilistic inference for general Bayesian networks. Hence we propose to view probabilistic inference in graphical models as a generic computational paradigm, that can help us to understand the computational organization of networks of neurons in the brain, and in particular the computational role of precisely structured cortical microcircuit motifs.

## Results

We present several ways how probabilistic inference for a given joint distribution 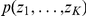, that is not required to have the form of a 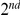 order Boltzmann distribution (5), can be carried out through sampling from the inherent dynamics of a recurrent network 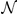 of stochastically spiking neurons. All these approaches are based on the idea that such network 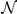 of spiking neurons can be viewed –for a suitable choice of its architecture and parameters –as an internal or “physical model” for the distribution 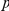, in the sense that its distribution of network states converges to 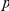, from any initial state. Then probabilistic inference for 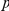 can be easily carried out by any readout neuron that observes the resulting network states, or the spikes from one or several neurons in the network. This holds not only for sampling from the prior distribution 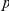, but also for sampling from the posterior after some evidence 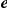 has become available (see (1)). The link between network states of 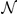 and the RVs 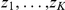 is provided by assuming that there exists for each RV 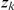 a neuron 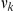 such that each time when 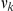 fires, it sets the associated binary RV 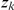 to 1 for a time period of some length 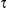 (see Fig. 1C). We refer to neurons 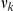 that represent in this way a RV 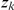 as principal neurons. All other neurons are referred to as auxiliary neurons.

The mathematical basis for analyzing the distribution of network states, and relating it to a given distribution 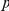, is provided by the theory of Markov chains. More precisely, it was shown in [1] that by introducing for each principal neuron 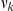 an additional hidden analog RV 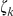, that keeps track of time within the time interval of length 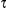 after a spike of 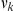, one can model the dynamics of the network 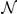 by a non-reversible Markov chain. This Markov chain is non-reversible, in contrast to Gibbs sampling or other Markov chains that are usually considered in Machine Learning and in the theory of Boltzmann machines, because this facilitates the modelling of the temporal dynamics of spiking neurons, in particular refractory processes within a spiking neuron after a spike and temporally extended effects of its spike on the membrane potential of other neurons to which it is synaptically connected (postsynaptic potentials). The underlying mathematical theory guarantees that nevertheless the distribution of network states of this Markov chain converges (for the “original” RVs 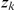) to the given distribution 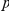, provided that the NCC (4) is met. This theoretical result reduces our goal, to demonstrate ways how a network of spiking neurons can carry out probabilistic inference in general graphical models, to the analysis of possibilities for satisfying the NCC (4) in networks of spiking neurons. The networks of spiking neurons that we construct and analyze build primarily on the model for neural sampling in continuous time from [1], since this continuous time version is the more satisfactory model from the biological perspective. But all our results also hold for the mathematically simpler version with discrete time.

We exhibit both methods for satisfying the NCC with the help of auxiliary neurons in networks of point neurons, and in networks of multi-compartment neuron models (where no auxiliary neurons are required). All neuron models that we consider are stochastic, where the probability density function for the firing of a neuron at time 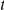 (provided it is currently not in a refractory state) is proportional to 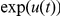, where 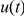 is its current membrane potential at the soma. We assume (as in [1]) that in a point neuron model the membrane potential 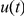 can be written as a linear combination of postsynaptic potentials. Thus if the principal neuron 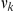 is modelled as a point neuron, we have
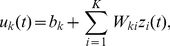
(8)where 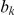 is the bias of neuron 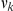 (which regulates its excitability), 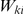 is the strength of the synaptic connection from neuron 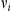 to 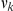, and 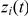 approximates the time course of the postsynaptic potential in neuron 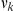 caused by a firing of neuron 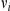. The ideal neuron model from the perspective of the theory of [1] has an absolute refractory period of length 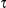, which is also the assumed length of a postsynaptic potential (EPSP or IPSP). But it was shown there through computer simulations that neural sampling can be carried out also with stochastically firing neurons that have a relative refractory period, i.e. the neuron can fire with some probability with an interspike interval of less than 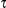. In particular, it was shown there in simulations that the resulting neural network samples from a slight variation of the target distribution 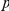, that is in most cases practically indistinguishable.

Before we describe two different theoretical approaches for satisfying the NCC, we first consider an even simpler method for extending the neural sampling approach from [1] to arbitrary distributions 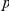: through a reduction to 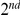 order Boltzmann distributions (5) with auxiliary RVs.

### Second Order Boltzmann Distributions with Auxiliary Random Variables (Implementation 1)

It is well known [15] that any probability distribution 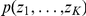, with arbitrarily large factors in a factorization such as (7), can be represented as marginal distribution
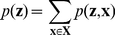
(9)of an extended distribution 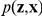 with auxiliary RVs 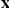, that can be factorized into factors of degrees at most 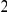. This can be seen as follows. Let 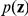 be an arbitrary probability distribution over binary variables with higher order factors 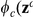). Thus
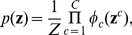
(10)where 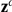 is a vector composed of the RVs that the factor 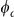 depends on and 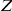 is a normalization constant. We additionally assume that 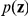 is non-zero for each value of 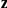. The simple idea is to introduce for each possible assignment 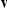 to the RVs 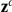 in a higher order factor 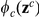 a new RV 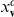, that has value 1 only if 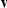 is the current assignment of values to the RVs in 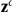. We will illustrate this idea through the concrete example of Fig. 1. Since there is only one factor that contains more than 2 RVs in the probability distribution of this example (see caption of Fig. 1), the conditional probability 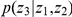, there will be 8 auxiliary RVs 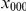, 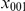, …, 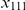 for this factor, one for each of the 8 possible assignments to the 3 RVs in 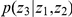. Let us consider a particular auxiliary RV, e.g. 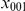. It assumes value 1 only if 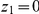, 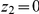, and 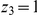. This constraint for 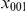 can be enforced through second order factors between 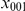 and each of the RVs 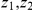 and 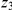. For example, the second order factor that relates 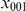 and 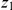 has a value of 0 if 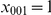 and 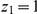 (i.e., if 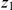 is not compatible with the assignment 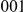), and value 1 otherwise. The individual values of the factor 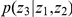 for different assignments to 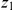, 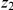 and 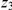 are introduced in the extended distribution 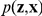 through first order factors, one for each auxiliary RV 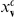. Specifically, the first order factor that depends on 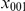 has value 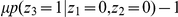 (where 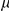 is a constant that rescales the values of the factors such that 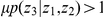 for all assignments to 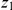, 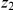 and 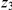) if 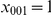, and value 1 otherwise. Further details of the construction method for 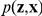 are given in the Methods section, together with a proof of (9).

The resulting extended probability distribution 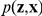 has the property that, in spite of deterministic dependencies between the RVs 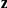 and 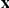, the state set of the resulting Markov chain realized through a network 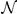 of spiking neurons according to [1] (that consists of all non-forbidden value assignments to 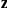 and 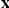) is connected. In the previous example a non-forbidden value assignment is 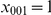 and 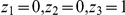. But 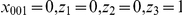 is also a non-forbidden value assignment. Such non-forbidden value assignments to the auxiliary RVs 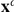 corresponding to one higher order factor, where all of them assume value of 0 regardless of the values of the 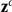 RVs provide transition points for paths of probability 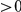 that connect any two non-forbidden value assignments (without requiring that 2 or more RVs switch their values simultaneously). The resulting connectivity of all non-forbidden states (see Methods for a proof) implies that this Markov chain has 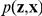 as its unique stationary distribution. The given distribution 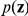 arises as marginal distribution of this stationary distribution of 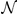 , hence one can use 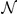 to sample from 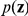 (just ignore the firing activity of neurons that correspond to auxiliary RVs 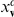).

Since the number of RVs in the extended probability distribution 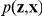 can be much larger than the number of RVs in 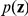, the corresponding spiking neural network samples from a much larger probability space. This, as well as the presence of deterministic relations between the auxiliary and the main RVs in the expanded probability distribution, slow down the convergence of the resulting Markov chain to its stationary distribution. We show however in the following, that there are several alternatives for sampling from an arbitrary distribution 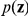 through a network of spiking neurons. These alternative methods do not introduce auxiliary RVs 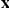, but rather aim at directly satisfying the NCC (4) in a network of spiking neurons. Note that the principal neurons in the neural network that implements neural sampling through introduction of auxiliary RVs 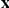 also satisfy the NCC, but in the extended probability distribution with second order relations 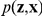, whereas in the neural implementations introduced in the following the principal neurons satisfy the NCC in the original distribution 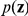. In Computer Simulation I we have compared the convergence speed of the methods that satisfy the NCC with that of the previously described method via auxiliary RVs. It turns out that the alternative strategy provides an about 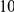 fold speed-up for the Bayesian network of Fig. 1B.

### Using the Markov Blanket Expansion of the Log-odd Ratio

Assume that the distribution 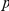 for which we want to carry out probabilistic inference is given by some arbitrary Bayesian network 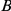. There are two different options for satisfying the NCC for 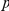, which differ in the way by which the term on the r.h.s. of the NCC (4) is expanded. The option that we will analyze first uses from the structure of the Bayesian network 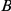 only the information about which RVs are in the Markov blanket of each RV 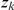. The Markov blanket 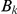 of the corresponding node 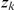 in 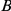 (which consists of the parents, children and co-parents of this node) has the property that 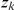 is independent from all other RVs once any assignment 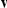 of values to the RVs 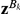 in the Markov blanket has been fixed. Hence 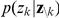 = 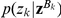, and the term on the r.h.s. of the NCC (4) can be expanded as follows:
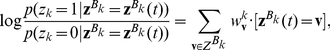
(11)where
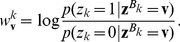
(12)The sum indexed by 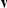 runs over the set 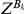 of all possible assignments of values to 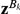, and 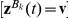 denotes a predicate which has value 1 if the condition in the brackets is true, and to 0 otherwise. Hence, for satisfying the NCC it suffices if there are auxiliary neurons, or dendritic branches, for each of these 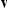, that become active if and only if the variables 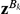 currently assume the value 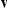. The current values of the variables 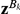 are encoded in the firing activity of their corresponding principal neurons. The corresponding term 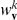 can be implemented with the help of the bias 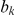 (see (8)) of the auxiliary neuron that corresponds to the assignment 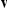, resulting in a value of its membrane potential equal to the r.h.s. of the NCC (4). We will discuss this implementation option below as Implementation 2. In the subsequently discussed implementation option (Implementation 3) all principal neurons will be multi-compartment neurons, and no auxiliary neurons are needed. In this case 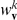 scales the amplitude of the signal from a specific dendritic branch to the soma of the multi-compartment principal neuron 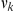.

#### Implementation with auxiliary neurons (Implementation 2)

**Figure 2:**
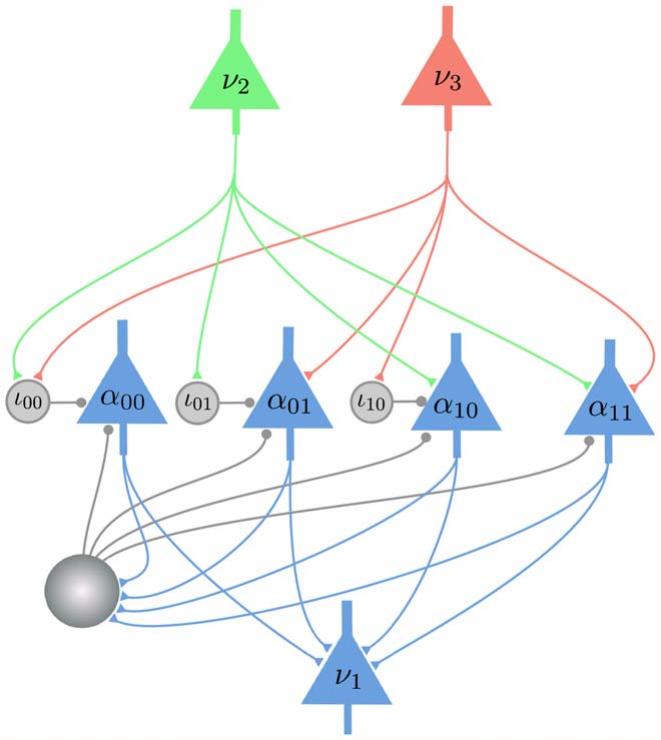
Implementation 2 for the explaining away motif of the Bayesian network from **Fig. 1B**. Implementation 2 is the neural implementation with auxiliary neurons, that uses the Markov blanket expansion of the log-odd ratio. There are 4 auxiliary neurons, one for each possible value assignment to the RVs 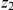 and 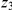 in the Markov blanket of 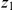. The principal neuron 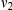 (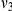) connects to the auxiliary neuron 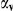 directly if 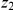 (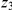) has value 1 in the assignment 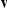, or via an inhibitory inter-neuron 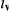 if 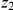 (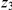) has value 0 in 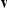. The auxiliary neurons connect with a strong excitatory connection to the principal neuron 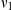, and drive it to fire whenever any one of them fires. The larger gray circle represents the lateral inhibition between the auxiliary neurons.

We illustrate the implementation of the Markov blanket expansion approach through auxiliary neurons for the concrete example of the RV 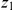 in the Bayesian network of Fig. 1B (see Methods for a discussion of the general case). Its Markov blanket 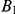 consists here of the RVs 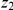 and 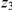. Hence the resulting neural circuit (see Fig. 2) for satisfying the NCC for the principal neuron 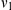 uses 4 auxiliary neurons 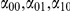 and 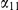, one for each of the 4 possible assignments 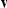 of values to the RVs 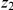 and 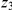. Each firing of one of these auxiliary neurons should cause an immediately subsequent firing of the principal neuron 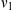. Lateral inhibition among these auxiliary neurons can make sure that after a firing of an auxiliary neuron no other auxiliary neuron fires during the subsequent time interval of length 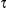, thereby implementing the required absolute refractory period of the theoretical model from [1]. The presynaptic principal neuron 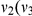) is connected to the auxiliary neuron 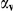 directly if 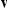 assumes that 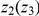 has value 1, otherwise via an inhibitory interneuron 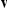 (see Fig. 2). In case of a synaptic connection via an inhibitory interneuron, a firing of 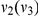 prevents a firing of this auxiliary neuron during the subsequent time interval of length 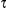. The direct excitatory synaptic connections from 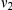 and 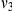 raise the membrane potential of that auxiliary neuron 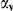, for which 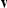 agrees with the current values of the RVs 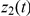 and 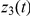, so that it reaches the value 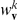, and fires with a probability equal to the r.h.s. of the NCC (4) during the time interval within which the value assignment 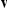 remains valid. The other 3 auxiliary neurons are during this period either inhibited by the inhibitory interneurons, or do not receive enough excitatory input from the direct connections to reach a significant firing probability. Hence, the principal neuron 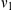 will always be driven to fire just by a single auxiliary neuron 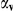 corresponding to the current value of the variables 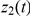 and 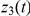, and will fire immediately after 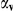 fires.

As 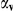 has a firing probability that satisfies the r.h.s. of the NCC (4) temporally during the time interval while 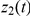 and 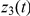 are consistent with 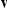, the firing of the principal neuron 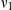 satisfies the r.h.s. of the NCC (4) at any moment in time.

#### Computer Simulation I: Comparison of two methods for emulating “explaining away” in networks of spiking neurons

**Figure 3:**
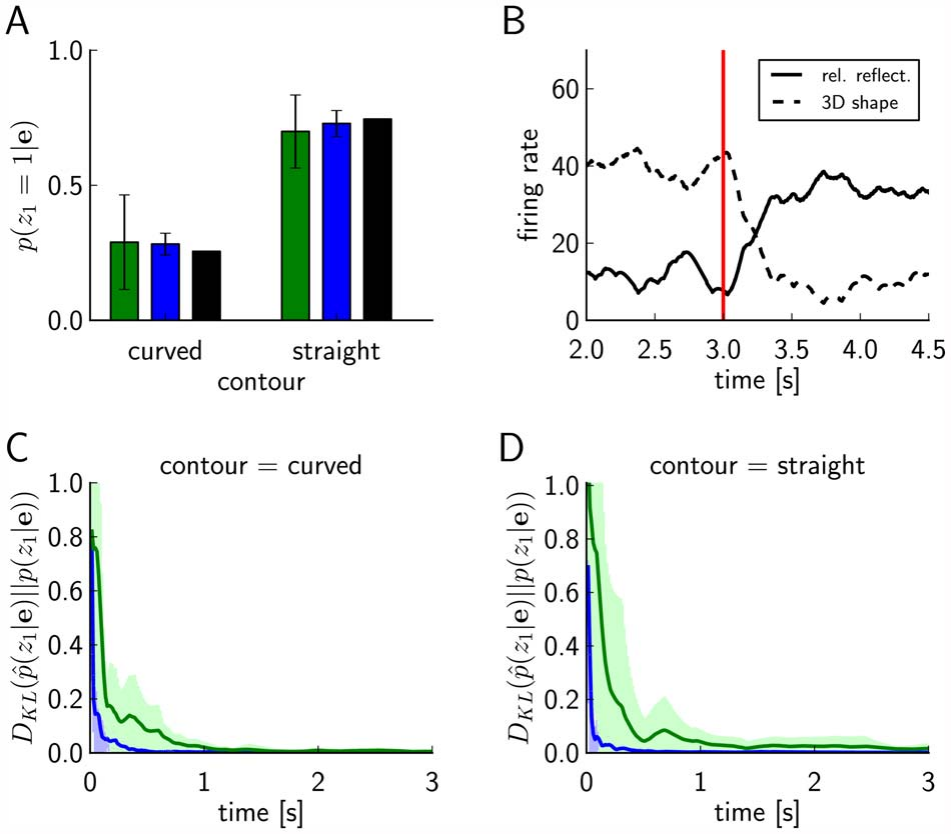
Results of Computer Simulation I. Performance comparison between an ideal version of Implementation 1 (use of auxiliary RVs, results shown in *green*) and an ideal version of implementations that satisfy the NCC (results shown in *blue*) for probabilistic inference in the Bayesian network of Fig. 1B (“explaining away”. Evidence 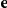 (see (1)) is entered for the RVs 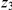 and 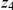, and the marginal probability 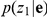 is estimated. **A**) Target values of 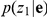 for 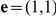 and 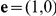 are shown in black, results from sampling for 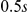 from a network of spiking neurons are shown in green and blue. Panels **C**) and **D**) show the temporal evolution of the Kullback-Leibler divergence between the resulting estimates through neural sampling 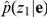 and the correct posterior 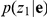, averaged over 10 trials for 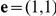 in C) and for 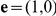 in D). The green and blue areas around the green and blue curves represent the unbiased value of the standard deviation. The estimated marginal posterior is calculated for each time point from the samples (number of spikes) from the beginning of the simulation (or from 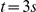 for the second inference query with 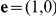). Panels A, C, D show that both approaches yield correct probabilistic inference through neural sampling, but the approach via satisfying the NCC converges about 10 times faster. **B**) The firing rates of principal neuron 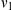 (solid line) and of the principal neuron 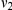 (dashed line) in the approach via satisfying the NCC, estimated with a sliding window (alpha kernel 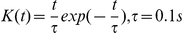). In this experiment the evidence 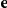 was switched after 3 s (red vertical line) from 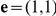 to 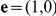. The “explaining away”effect is clearly visible from the complementary evolution of the firing rates of the neurons 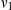 and 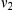.

In our preceding theoretical analysis we have exhibited two completely different methods for emulating in networks of spiking neurons probabilistic inference in general graphical models through sampling: either by a reduction to 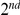 order Boltzmann distributions (5) through the introduction of auxiliary RVs (Implementation 1), or by satisfying the NCC (3) via the Markov blanket expansion. We have tested the accuracy and convergence speed of both methods for the Bayesian network of Fig. 1B, and the results are shown in Fig. 3. The approach via the NCC converges substantially faster.

#### Implementation with dendritic computation (Implementation 3)

**Figure 4:**
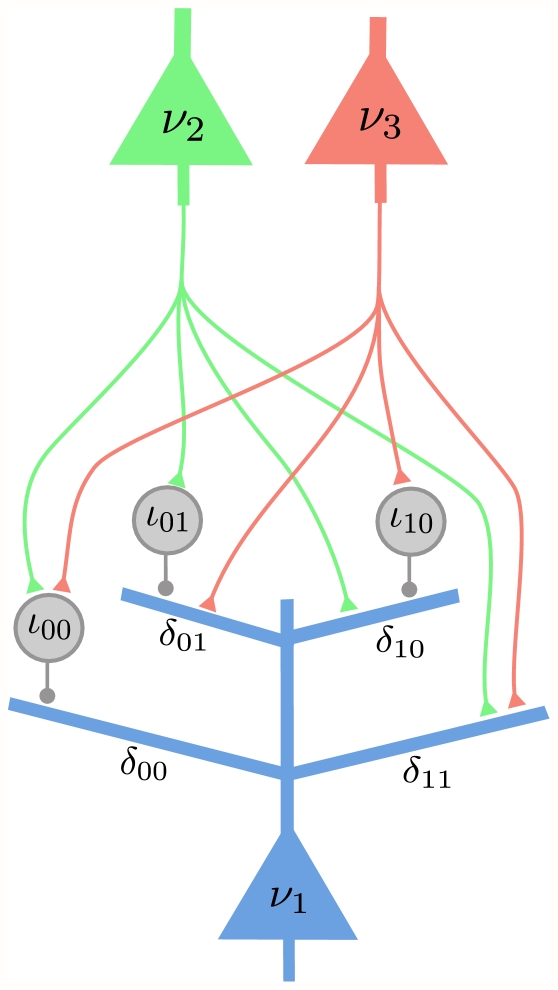
Implementation 3 for the same explaining away motif as in **Fig. 2**. Implementation 3 is the neural implementation with dendritic computation that uses the Markov blanket expansion of the log-odd ratio. The principal neuron 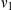 has 4 dendritic branches, one for each possible assignment of values 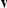 to the RVs 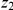 and 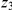 in the Markov blanket of 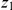. The dendritic branches of neuron 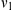 receive synaptic inputs from the principal neurons 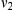 and 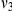 either directly, or via an interneuron (analogously as in Fig. 2). It is required that at any moment in time exactly one of the dendritic branches (that one, whose index 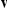 agrees with the current firing states of 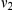 and 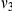) generates dendritic spikes, whose amplitude at the soma determines the current firing probability of 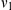.

We now show that the Markov blanket expansion approach can also be implemented through dendritic branches of multi-compartment neuron models (see Methods) for the principal neurons, without using auxiliary neurons (except for inhibitory interneurons). We will illustrate the idea through the same Bayesian network example as discussed in Implementation 2, and refer to Methods for a discussion of the case of arbitrary Bayesian networks. Fig. 4 shows the principal neuron 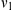 in the spiking neural network for the Bayesian network of Fig. 1B. It has 4 dendritic branches 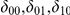 and 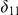, each of them corresponding to one assignment 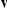 of values to the variables 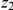 and 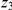 in the Markov blanket of 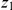. The input connections from the principal neurons 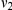 and 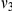 to the dendritic branches of 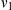 follow the same pattern as the connections from 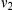 and 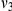 to the auxiliary neurons in Implementation 2. Let 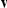 be an assignment that corresponds to the current values of the variables 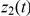 and 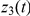. The efficacies of the synapses at the dendritic branches and their thresholds for initiating a dendritic spike are chosen such that the total synaptic input to the dendritic branch 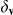 is then strong enough to cause a dendritic spike in the branch, that contributes to the membrane potential at the soma a component whose amplitude is equal to the parameter 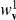 in (11). This amplitude could for example be controlled by the branch strength of this dendritic branch (see [22], [23]). The parameters can be chosen so that all other dendritic branches do not receive enough synaptic input to reach the local threshold for initiating a dendritic spike, and therefore do not affect the membrane potential at the soma. Hence, the membrane potential at the soma of 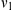 will be equal to the contribution from the currently active dendritic branch 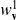, implementing thereby the r.h.s of (11).

Since the parameters 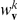 in (11) can have both positive and negative values and the amplitude of the dendritic spikes and the excitatory synaptic efficacy are positive quantities, in this, and the following neural implementations we always add a positive constant to 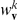 to shift it into the positive range. We subtract the same constant value from the steady state of the membrane potential.

### Using the Factorized Expansion of the Log-odd Ratio

**Figure 5 pcbi-1002294-g005:**
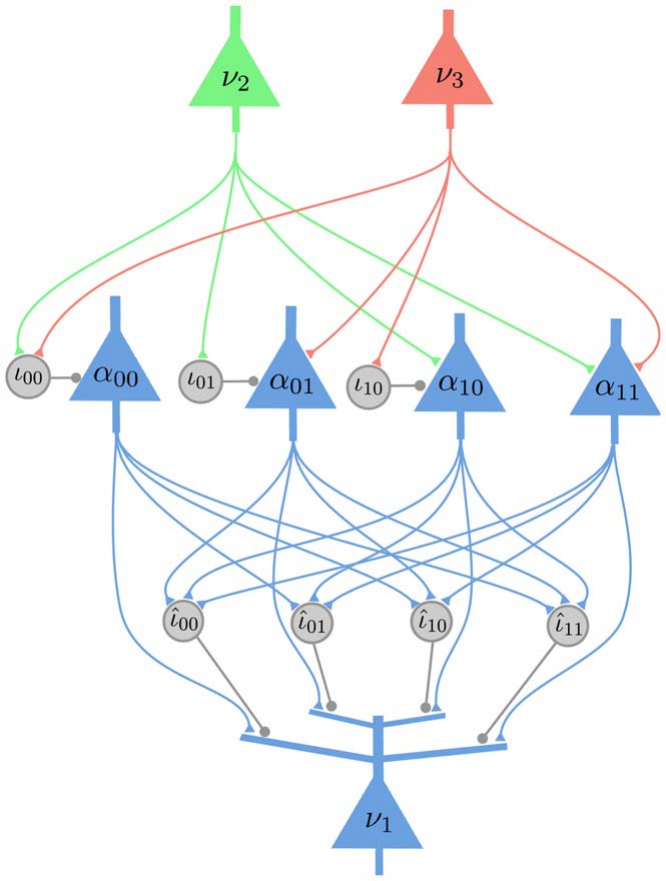
Implementation 4 for the same explaining away motif as in **Fig. 2** and **4**. Implementation 4 is the neural implementation with auxiliary neurons and dendritic branches, that uses the factorized expansion of the log-odd ratio. As in Fig. 2 there is one auxiliary neuron 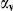 for each possible value assignment 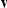 to 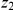 and 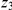. The connections from the neurons 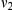 and 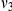 (that carry the current values of the RVs 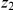 and 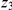) to the auxiliary neurons are the same as in Fig. 2, and when these RVs change their value, the auxiliary neuron that corresponds to the new value fires. Each auxiliary neuron 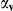 connects to the principal neuron 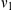 at a separate dendritic branch 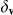, and there is an inhibitory neuron 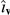 connecting to the same branch. The rest of the auxiliary neurons connect to the inhibitory interneuron 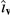. The function of the inhibitory neuron 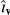 is to shunt the active EPSP caused by a recent spike from the auxiliary neuron 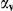 when the value of the 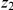 and 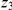 changes from 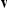 to another value.

The second strategy to expand the log-odd ratio on the r.h.s. of the NCC (4) uses the factorized form (10) of the probability distribution 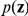. This form allows us to rewrite the log-odd ratio in (4) as a sum of log terms, one for each factor 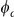, 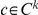, that contains the RV 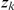 (we write 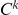 for this set of factors). One can write each of these terms as a sum over all possible assignments 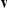 of values of the variables 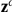 the factor 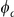 depends on (except 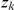). This yields
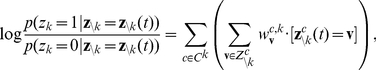
(13)where 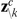 is a vector composed of the RVs 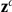 that the factor 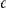 depends on –without 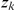, and 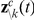 is the current value of this vector at time 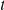. 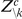 denotes the set of all possible assignments to the RVs 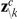. The parameters 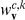 are set to
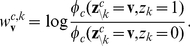
(14)The factorized expansion in (13) is similar to (11), but with the difference that we have another sum running over all factors that depend on 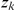. Consequently, in the resulting Implementation 4 with auxiliary neurons and dendritic branches there will be several groups of auxiliary neurons that connect to 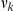, where each group implements the expansion of one factor in (13). The alternative model that only uses dendritic computation (Implementation 5) will have groups of dendritic branches corresponding to the different factors. The number of auxiliary neurons that connect to 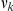 in Implementation 4 (and the corresponding number of dendritic branches in Implementation 5) is equal to the sum of the exponents of the sizes of factors that depend on 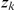: 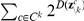, where 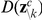 denotes the number of RVs in the vector 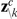. This number is never larger than 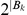 (where 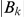 is the size of the Markov blanket of 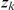), which gives the corresponding number of auxiliary neurons or dendritic branches that are required in the Implementation 2 and 3. These two numbers can considerably differ in graphical models where the RVs participate in many factors, but the size of the factors is small. Therefore one advantage of this approach is that it requires in general fewer resources. On the other hand, it introduces a more complex connectivity between the auxiliary neurons and the principal neuron (compare Fig. 5 with Fig. 2).

#### Implementation with auxiliary neurons and dendritic branches (Implementation 4)

A salient difference to the Markov blanket expansion and Implementation 2 arises from the fact that the r.h.s. of the factor expansion (13) contains an additional summation over all factors 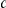 that contain the RV 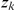. This entails that the principal neuron 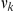 has to sum up inputs from several groups of auxiliary neurons, one for each factor 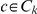. Hence in contrast to Implementation 2, where the principal neuron fired whenever one of the associated auxiliary neurons fired, we now aim at satisfying the NCC by making sure that the membrane potential of 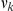 approximates at any moment in time the r.h.s. of the NCC (4). One can achieve this by making sure that each auxiliary neuron 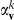 fires immediately when the presynaptic principal neurons assume state 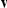 and by having a synaptic connection between 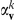 and 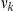 with a synaptic efficacy equal to 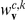 from (13). Some imprecision of the sampling may arise when the value of variables in 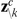 changes, while EPSPs caused by an earlier value of these variables have not yet vanished at the soma of 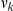. This problem can be solved if the firing of the auxiliary neuron caused by the new value of 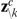 shunts such EPSP, that had been caused by the preceding value of 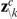, directly in the corresponding dendrite. This shunting inhibition should have minimal effect on the membrane potential at the soma of 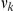. Therefore excitatory synaptic inputs from different auxiliary neurons 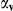 (that cause a depolarization by an amount 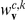 at the soma) should arrive on different dendritic branches 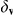 of 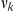 (see Fig. 5), that also have connections from associated inhibitory neurons 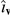.

Fig. 5 shows the resulting implementation for the same explaining away motif of Fig. 1B as the preceding figures 2 and 4. Note that the RV 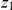 occurs there only in a single factor 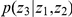, such that the previously mentioned summation of EPSPs from auxiliary neurons that arise from different factors cannot be demonstrated in this example.

#### Implementation with dendritic computation (Implementation 5)

**Figure 6:**
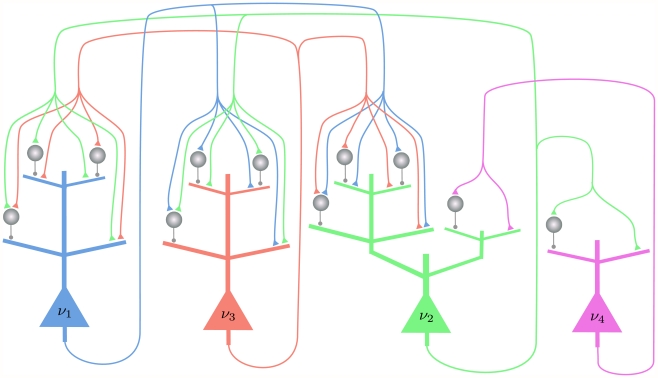
Implementation 5 for the Bayesian network shown in **Fig. 1B**. Implementation 5 is the implementation with dendritic computation that is based on the factorized expansion of the log-odd ratio. RV 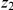 occurs in two factors, 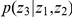 and 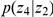, and therefore 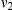 receives synaptic inputs from 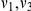 and 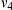 on separate groups of dendritic branches. Altogether the synaptic connections of this network of spiking neurons implement the graph structure of Fig. 1D.

The last neural implementation that we consider is an adaptation of Implementation 3 (the implementation with dendritic computation, that uses the Markov blanket expansion of the log-odd ratio) to the factorized expansion of the log-odd ratio. In this case each principal neuron, instead of having all its dendritic branches corresponding to different value assignments to the RVs of the Markov blanket, has several groups of dendritic branches, where each group corresponds to the linear expansion of one factor in the log-odd ratio in (13). Fig. 6 shows the complete spiking neural network that samples from the Bayesian network of Fig. 1B. The principal neuron 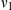 has the same structure and connectivity as in Implementation 3 (see Fig. 4), since the RV 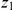 participates in only one factor, and the set of variables other than 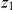 in this factor constitute the Markov blanket of 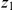. The same is true for the principal neurons 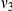 and 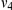. As the RV 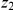 occurs in two factors, the principal neuron 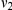 has two groups of dendritic branches, 4 for the factor 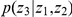 with synaptic input from the principal neurons 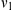 and 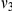, and 2 for the factor 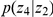 with synaptic inputs from the principal neuron 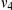. Note for comparison, that this neuron 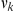 needs to have 8 dendritic branches in Implementation 3, one for each assignment of values to the variables 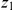, 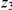 and 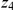 in the Markov blanket of 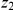.

The number of dendritic branches of a principal neuron 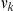 in this implementation is the same as the number of auxiliary neurons for 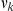 in Implementation 4, and is never larger than the number of dendritic branches of the neuron 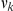 in Implementation 3. Although this implementation is more efficient with respect to the required number of dendritic branches, when considering the possible application of STDP for learning in Implementation 3, it has the advantage that it could learn an approximate generative model of the probability distribution of the inputs without knowing apriori the factorization of the probability distribution.

The amplitude of the dendritic spikes from the dendritic branch 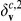 of the principal neuron 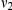 should be equal to the parameter 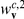 from (13). The index 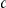 identifies the two factors that depend on 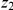. The membrane voltage at the soma of the principal neuron 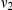 is then equal to the sum of the contributions from the dendritic spikes of the active dendritic branches. At time 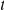 there is exactly one active branch in each of the two groups of dendritic branches. The sum of the contributions from the two active dendritic branches results in a membrane voltage at the soma of the principal neuron that corresponds to the r.h.s of the (13). In the Methods section we provide a general and detailed explanation of this approach.

### Probabilistic Inference through Neural Sampling in Larger and More Complex Bayesian Networks

We have tested the viability of the previously described approach for neural sampling by satisfying the NCC also on two larger and more complex Bayesian networks: the well-known ASIA-network [24], and an even larger randomly generated Bayesian network. The primary question is in both cases, whether the convergence speed of neural sampling is in a range where a reasonable approximation to probabilistic inference can be provided within the typical range of biological reaction times of a few 100 ms. In addition, we examine for the ASIA-network the question to what extent more complex and biologically more realistic shapes of EPSPs affect the performance. For the larger random Bayesian network we examine what difference in performance is caused by neuron models with absolute versus relative refractory periods.

#### Computer Simulation II: ASIA Bayesian network

**Figure 7:**
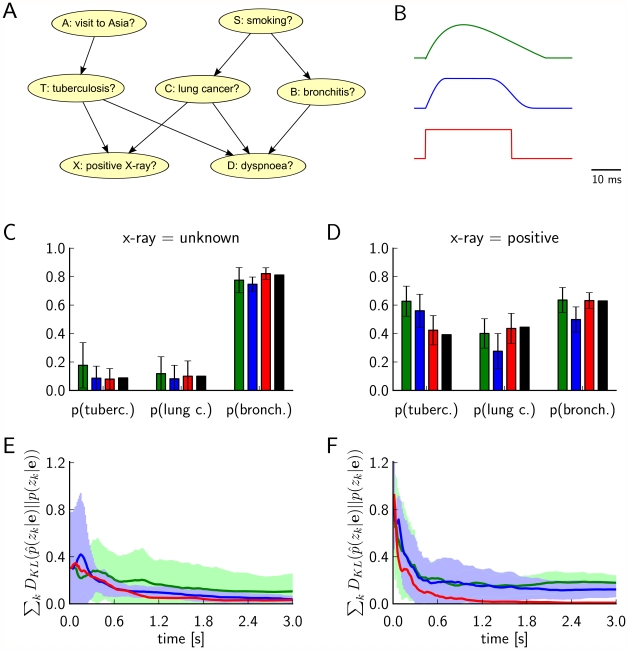
Results of Computer Simulation II. Probabilistic inference in the ASIA network with networks of spiking neurons that use different shapes of EPSPs. The simulated neural networks correspond to Implementation 2. The evidence is changed at 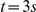 from 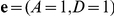 to 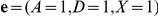 (by clamping the x-ray test RV to 1). The probabilistic inference query is to estimate marginal posterior probabilities 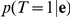, 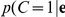, and 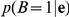. **A**) The ASIA Bayesian network. **B**) The three different shapes of EPSPs, an alpha shape (green curve), a smooth plateau shape (blue curve) and the optimal rectangular shape (red curve). **C**) and **D**) Estimated marginal probabilities for each of the diseases, calculated from the samples generated during the first 800 ms of the simulation with alpha shaped (green bars), plateau shaped (blue bars) and rectangular (red bars) EPSPs, compared with the corresponding correct marginal posterior probabilities (black bars), for 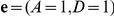 in C) and 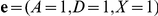 in D). The results are averaged over 20 simulations with different random initial conditions. The error bars show the unbiased estimate of the standard deviation. **E**) and **F**) The sum of the Kullback-Leibler divergences between the correct and the estimated marginal posterior probability for each of the diseases using alpha shaped (green curve), plateau shaped (blue curve) and rectangular (red curve) EPSPs, for 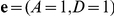 in E) and 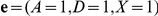 in F). The results are averaged over 20 simulation trials, and the light green and light blue areas show the unbiased estimate of the standard deviation for the green and blue curves respectively (the standard deviation for the red curve is not shown). The estimated marginal posteriors are calculated at each time point from the gathered samples from the beginning of the simulation (or from 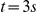 for the second inference query with 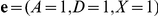).

The ASIA-network is an example for a larger class of Bayesian networks that are of special interest from the perspective of Cognitive Science [25]. Networks of this type, that consist of 3 types of RVs (context information, true causes, observable symptoms) with directed edges only from one class to the next, capture the causal structure behind numerous domains of human reasoning. The ASIA-network (see Fig. 7A) encodes knowledge about direct influences between environmental factors, 3 specific diseases, and observable symptoms. A concrete distribution 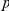 that is compatible with this Bayesian network was specified through conditional probabilities for each node as in [24] (with one small change to avoid deterministic relationship among RVs, see Methods). The binary RVs of the network encode whether a person had a recent visit to Asia (A), whether the person smokes (S), the presence of diseases tuberculosis (T), lung cancer (C), and bronchitis (B), the presence of the symptom dyspnoea (D), and the result of a chest x-ray test (X). This network not only contains multiple “explaining away” effects (i.e., nodes with more than one parent), but also a loop (i.e., undirected cycle) between the RVs S, B, D, C. Hence no probabilistic inference approach based on belief propagation executed directly on this ASIA Bayesian network is guaranteed to work.

A typical example for probabilistic inference in this network arises when one enters as evidence the facts that the patient visited Asia (A = 1) and has Dyspnoea (D = 1), and asks what is the likelihood of each of the RVs T, C, B that represent the diseases, and how the result of a positive x-ray test would affects these likelihoods.

We tested this probabilistic inference in a network of spiking neurons according to Implementation 2 with three different shapes of the EPSPs: an alpha EPSP, a plateau EPSP and the optimal rectangular EPSP (See Fig. 7B). These shapes match qualitatively the shapes of EPSPs recorded in the soma of pyramidal neurons for synaptic inputs that arrive on dendritic branches (see Fig. 1 in [26]). The neurons in the spiking neural network had an absolute refractory period. Fig. 7C, D show that the network provides for all three shapes of the EPSPs within 800 ms of simulated biological time quite accurate answers to the tested probabilistic inference query. Fig. 7E, F show that also with smoother shapes of the EPSPs the networks arrive at good heuristic answers within several hundreds of milliseconds. The Kullback-Leibler divergence converges in this case to a small non-zero value, indicating an error caused by the non-ideal sampling process.

**Figure 8:**
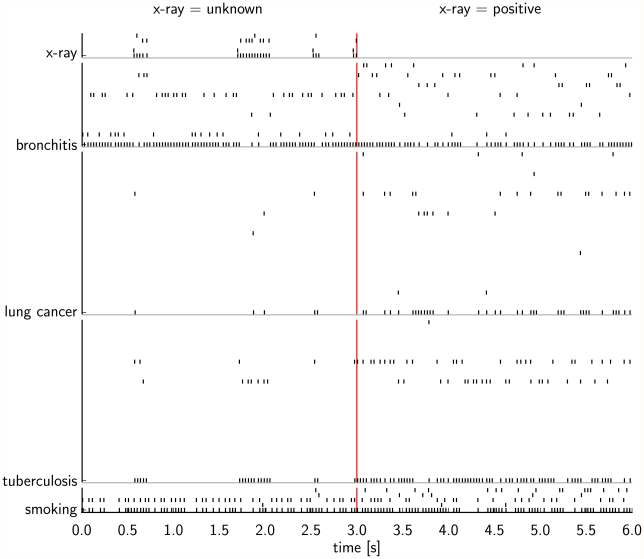
Spike raster of the spiking activity in one of the simulation trials described in **Fig. 7**. The spiking activity is from a simulation trial with the network of spiking neurons with alpha shaped EPSPs. The evidence was switched after 3 s (red vertical line) from 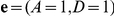 to 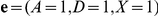 (by clamping the RV X to 1). In each block of rows the lowest spike train shows the activity of a principal neuron (see left hand side for the label of the associated RV), and the spike trains above show the firing activity of the associated auxiliary neurons. After 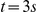 the activity of the neurons for the x-ray test RV is not shown, since during this period the RV is clamped and the firing rate of its principal neuron is induced externally.

Fig. 8 shows the spiking activity of the neural network with alpha shaped EPSPs in one of the simulation trials. During the first 3 seconds of the simulation the network alternated between two different modes of spiking activity, that correspond to two different modes of the posterior probability distribution. There are time periods when the principal neuron for the RV X (positive X-ray), T (tuberculosis) and C (lung c.) had a higher firing rate, with time periods in between where they were silent. After 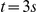, when the evidence that the x-ray test is positive was introduced, the activity of the network remained in the first mode.

#### Computer Simulation III: Randomly generated Bayesian network

**Figure 9:**
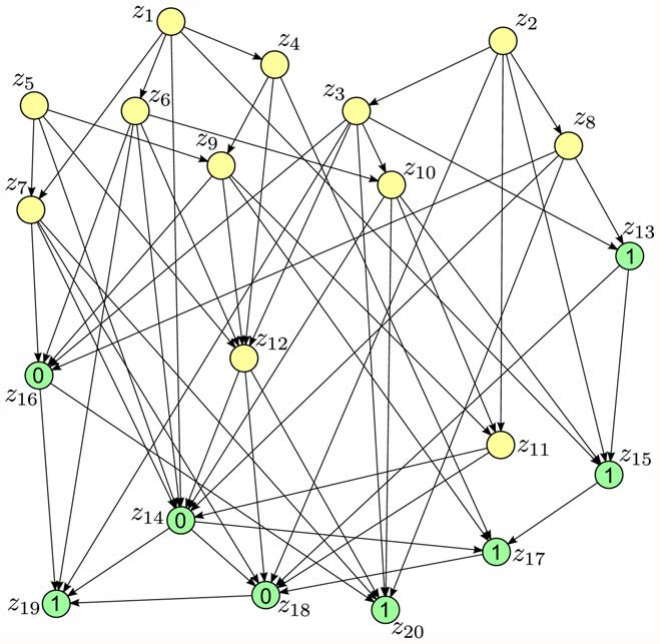
The randomly generated Bayesian network used in Computer Simulation III. It contains 20 nodes. Each node has up to 8 parents. We consider the generic but more difficult instance for probabilistic inference where evidence 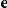 is entered for nodes 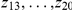 in the lower part of the directed graph. The conditional probability tables were also randomly generated for all RVs.

In order to test the performance of neural sampling for an “arbitrary” less structured, and larger graphical model, we generated a random Bayesian network according to the method proposed in [27] (the details of the generation algorithm are given in the Methods section). We added an additional constraint, that the maximum in-degree of the nodes should be not larger than 8. A resulting randomly generated network is shown in Fig. 9. It contains nodes with up to 8 parents, and it also contains numerous loops. For the RVs 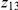 to 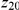 we fixed a randomly chosen assignment 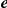. Neural sampling was tested for an ideal neural network that satisfies the NCC with a variety of random initial states, using spiking neurons with an absolute, and alternatively also with a relative refractory period.

**Figure 10:**
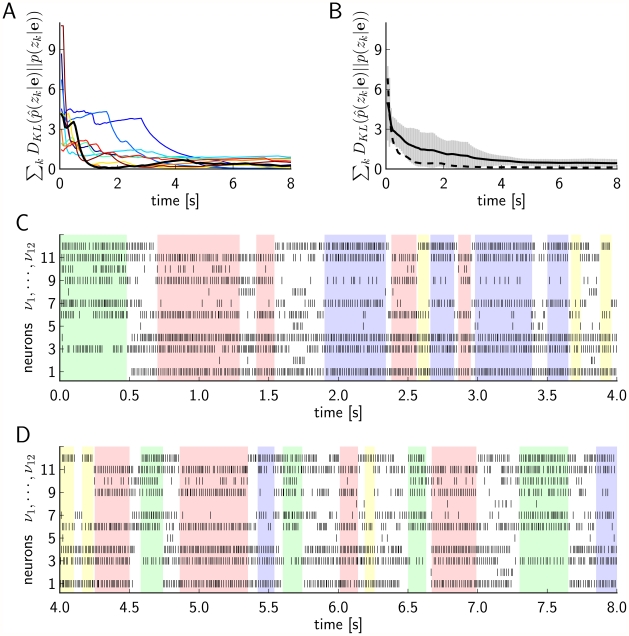
Results of Computer Simulation III. Neural emulation of probabilistic inference through neural sampling in the fairly large and complex randomly chosen Bayesian network shown in Fig. 9. **A**) The sum of the Kullback-Leibler divergences between the correct and the estimated marginal posterior probability for each of the unobserved random variables 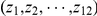, calculated from the generated samples (spikes) from the beginning of the simulation up to the current time indicated on the x-axis, for simulations with a neuron model with relative refractory period. Separate curves with different colors are shown for each of the 10 trials with different initial conditions (randomly chosen). The bold black curve corresponds to the simulation for which the spiking activity is shown in C) and D). **B**) As in A) but the mean over the 10 trials is shown, for simulations with a neuron model with relative refractory period (solid curve) and absolute refractory period (dashed curve.). The gray area around the solid curve shows the unbiased estimate of the standard deviation calculated over the 10 trials. **C**) and **D**) The spiking activity of the 12 principal neurons during the simulation from 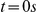 to 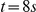, for one of the 10 simulations (neurons with relative refractory period). The neural network enters and remains in different network states (indicated by different colors), corresponding to different modes of the posterior probability distribution.

Fig. 10A shows that in most of our 10 simulations (with different randomly chosen initial states and different random noise throughout the simulation) the sum of Kullback-Leibler divergences for the 12 RVs 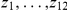 becomes quite small within a second. Only in a few trials several seconds were needed for that. Fig. 10C and 10D show the spiking activity of the neural network from 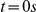 to 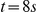 in one of the 10 trials. It is interesting to observe that the network went through a number of network states, each of them characterized by a high firing rate of a particular subset of the neurons.

Similarly spontaneous switchings between internal network states have been reported in numerous biological experiments (see e.g. [28], [29]), but their functional role has remained unknown. In the context of Computer Simulation III these switchings between network states arise because this is the only way how this network of spiking neurons can sample from a multi-modal target distribution 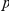.

## Discussion

We have shown through rigorous theoretical arguments and computer simulations that networks of spiking neurons are in principle able to emulate probabilistic inference in general graphical models. The latter has emerged as a quite suitable mathematical framework for describing those computational tasks that artificial and biological intelligent agents need to solve. Hence the results of this article provide a link between this abstract description level of computational theory and models for networks of neurons in the brain. In particular, they provide a principled framework for investigating how nonlinear computational operations in network motifs of cortical microcircuits and in the dendritic trees of neurons contribute to brain computations on a larger scale. Altogether we view our approach as a contribution to the solution of a fundamental open problem that has been raised in Cognitive Science:

“What approximate algorithms does the mind use, how do they relate to engineering approximations in probabilistic AI, and how are they implemented in neural circuits? Much recent work points to Monte Carlo or stochastic sampling–based approximations as a unifying framework for understanding how Bayesian inference may work practically across all these levels, in minds, brains, and machines ” [13].

We have presented three different theoretical approaches for extending the results of [1], such that they yield explanations how probabilistic inference in general graphical models could be carried out through the inherent dynamics of recurrent networks of stochastically firing neurons (neural sampling). The first and simplest one was based on the fact that any distribution can be represented as marginal distribution of a 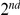 order Boltzmann distribution (5) with auxiliary RVs. However, as we have demonstrated in Fig. 3, this approach yields rather slow convergence of the distribution of network states to the target distribution. This is a natural consequence of the deterministic definition of new RVs in terms of the original RVs, which reduces the conductance [9], [30] (i.e., the probability to get from one set of network states to another set of network states) of the Markov chain that is defined by the network dynamics. Further research is needed to clarify whether this deficiency can be overcome through other methods for introducing auxiliary RVs.

We have furthermore presented two approaches for satisfying the NCC (3) of [1], which is a sufficient condition for sampling from a given distribution. These two closely related approaches rely on different ways of expanding the term on the r.h.s. of the NCC (4). The first approach can be used if the underlying graphical model implies that the Markov blankets of all RVs are relatively small. The second approach yields efficient neural emulations under a milder constraint: if each factor in a factorization of the target distribution is rather small (and if there are not too many factors). Each of these two approaches provides the theoretical basis for two different methods for satisfying the NCC in a network of spiking neurons: either through nonlinear computation in network motifs with auxiliary spiking neurons (that do not directly represent a RV of the target distribution), or through dendritic computation in multi-compartment neuron models. This yields altogether four different options for satisfying the NCC in a network of spiking neurons. These four options are demonstrated in Fig. 2, 4–6 for a characteristic explaining away motif in the simple Bayesian network of Fig. 1B, that had previously been introduced to model inference in biological visual processing [21]. The second approach for satisfying the NCC never requires more auxiliary neurons or dendritic branches than the first approach.

Each of these four options for satisfying the NCC would be optimally supported by somewhat different features of the interaction of excitation and inhibition in canonical cortical microcircuit motifs, and by somewhat different features of dendritic computation. Sufficiently precise and general experimental data are not yet available for many of these features, and we hope that the computational consequences of these features that we have exhibited in this article will promote further experimental work on these open questions. In particular, the neural circuit of Fig. 5 uses an implementation strategy that requires for many graphical models (those where Markov blankets are substantially larger than individual factors) fewer auxiliary neurons. But it requires temporally precise local inhibition in dendritic branches that has negligible effects on the membrane potential at the soma, or in other dendritic branches that are used for this computation. Some experimental results in this direction are reported in [31], where it was shown (see e.g. their Fig. 1) that IPSPs from apical dendrites of layer 5 pyramidal neurons are drastically attenuated at the soma. The options that rely on dendritic computation (Fig. 4 and 6) would be optimally supported if EPSPs from dendritic branches that are not amplified by dendritic spikes have hardly any effect on the membrane potential at the soma. Some experimental results which support this assumption for distal dendritic branches of layer 5 pyramidal neurons had been reported in [26], see e.g. their Fig. 1. With regard to details of dendritic spikes, these would optimally support the ideal theoretical models with dendritic computation if they would have a rather short duration at the soma, in order to avoid that they still affect the firing probability of the neuron when the state (i.e., firing or non-firing within the preceding time interval of length 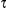) of presynaptic neurons has changed. In addition, the ideal impact of a dendritic spike on the membrane potential at the soma would approximate a step function (rather than a function with a pronounced peak at the beginning).

Another desired property of the dendritic spikes in context of our neural implementations is that their propagation from the dendritic branch to the soma should be very fast, i.e. with short delays that are much smaller than the duration of the EPSPs. This is in accordance with the results reported in [32] where they found (see their Fig. 1) that the fast active propagation of the dendritic spike towards the soma reduces the rise time of the voltage at the soma to less than a millisecond, in comparison to the 3 ms rise time during the propagation of the individual EPSPs when there is no dendritic spike. Further, in [22] it is shown that the latency of an action potential evoked by a strong dendritic spike, calculated with respect to the time of the activation of the synaptic input at the dendritic branch, is slightly below 2 ms, supporting the assumption of fast propagation of the dendritic spike to the soma.

We have focused in this article on the description of ideal neural emulations of probabilistic inference in general graphical models. These ideal neural implementations use a complete representation of the conditional odd-ratios, i.e. have a separate auxiliary neuron or dendritic branch for each possible assignment of values to the RVs in the Markov blanket in implementations 2 and 3, or in the factor in implementations 4 and 5. Hence, the required number of neurons (or dendritic branches) scales exponentially with the sizes of the Markov blankets and the factors in the probability distribution, and it would quickly become unfeasible to represent probability distributions with larger Markov blankets or factors. One possible way to overcome this limitation is to consider an approximate implementation of the NCC with fewer auxiliary neurons or dendritic branches. In fact, such an approximate implementation of the NCC could be learned. Our results provide the basis for investigating in subsequent work how approximations to these ideal neural emulations could emerge through synaptic plasticity and other adaptive processes in neurons. First explorations of these questions suggest that in particular approximations to Implementations 1,2 and 4 could emerge through STDP in a ubiquitous network motif of cortical microcircuits [33]: Winner-Take-All circuits formed by populations of pyramidal neurons with lateral inhibition. This learning-based approach relies on the observation that STDP enables pyramidal neurons in the presence of lateral inhibition to specialize each on a particular pattern of presynaptic firing activity, and to fire after learning only when this presynaptic firing pattern appears [34]. These neurons would then assume the role of the auxiliary neurons, both in the first option with auxiliary RVs, and in the options shown in Fig. 2 and 5. Furthermore, the results of [23] suggest that STDP in combination with branch strength potentiation enables individual dendritic branches to specialize on particular patterns of presynaptic inputs, similarly as in the theoretically optimal constructions of Fig. 4 and 6. One difference between the theoretically optimal neural emulations and learning based approximations is that auxiliary neurons or dendritic branches learn to represent only the most frequently occurring patterns of presynaptic firing activity, rather than creating a complete catalogue of all theoretically possible presynaptic firing patterns. This has the advantage that fewer auxiliary neurons and dendritic branches are needed in these biologically more realistic learning-based approximations.

Other ongoing research explores neural emulations of probabilistic inference for non-binary RVs. In this case a stochastic principal neuron 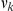 that represents a binary RV 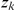 is replaced by a Winner-Take-All circuit, that encodes the value of a multinomial or analog RV through population coding, see [34].

### Related Work

There are a number of studies proposing neural network architectures that implement probabilistic inference [15], [17], [18], [35]–[48]. Most of these models propose neural emulations of the belief propagation algorithm, where the activity of neurons or populations of neurons encodes intermediate values (called messages or beliefs) needed in the arithmetical calculation of the posterior probability distribution. With some exceptions [17], most of the approaches assume rate-based coding of information and use rate-based neuron models or mean-field approximations.

In particular, in [37] a spiking neural network model was developed that performs the max-product message passing algorithm, a variant of belief propagation, where the necessary maximization and product operations were implemented by specialized neural circuits. Another spiking neural implementation of the sum-product belief propagation algorithm was proposed in [36], where the calculation and passing of the messages was achieved in a recurrent network of interconnected liquid state machines [49]. In these studies, that implemented probabilistic inference with spiking neurons through emulation of the belief propagation algorithm on tree factor graphs, the beliefs or the messages during the calculation of the posterior distributions were encoded in an average firing rate of a population of neurons. Regarding the complexity of these neural models, as the number of required computational operations in belief propagation is exponential in the size of the largest factor in the probability distribution, in the neural implementations this translates to a number of neurons in the network that scales exponentially with the size of the largest factor. This complexity corresponds to the required number of neurons (or dendritic branches) in implementations 1, 3 and 5 in our approach, whereas implementations 2 and 4 require a larger number of neurons that scales exponentially with the size of the largest Markov blanket in the distribution. Additionally, note that the time of convergence to the correct posterior differs in both approaches: in the belief propagation based models it scales in the worst case linearly with the number of RVs in the probability distribution, whereas in our approach it can vary depending on the probability distribution.

Although the belief propagation algorithm can be applied to graphical models with undirected loops (a variant called loopy belief propagation), it is not always guaranteed to work, which limits the applicability of the neural implementations based on this algorithm. The computation and the passing of messages in belief propagation uses, however, equivalent computations as the junction tree algorithm [24], [50], a message passing algorithm that operates on a junction tree, a tree structure derived from the graphical model. The junction tree algorithm performs exact probabilistic inference in general graphical models, including those that have loops. Hence, the neural implementations of belief propagation could in principle be adapted to work on junction trees as well. This however comes at a computational cost manifested in a larger required size of the neural network, since the number of required operations for the junction tree algorithm scales exponentially with the width of the junction tree, and the width of the junction tree can be larger than the size of the largest factor for graphical models that have loops (see [9], chap. 10 for a discussion). The analysis of the complexity and performance of resulting emulations in networks of spiking neurons is an interesting topic for future research.

Another interesting approach, that adopts an alternative spike-time based coding scheme, was described in [17]. In this study a spiking neuron model estimates the log-odd ratio of a hidden binary state in a hidden Markov model, and it outputs a spike only when it receives new evidence from the inputs that causes a shift in the estimated log-odd ratio that exceeds a certain threshold, that is, only when new information about a change in the log-odd ratio is presented that cannot be predicted by the preceding spikes of the neuron. However, this study considers only a very restricted class of graphical models: Bayesian networks that are trees (where for example no explaining away can occur). The ideas in [17] have been extended in [18], where the neural model is capable of integration of evidence from multiple simultaneous cues (the underlying graphical model is a hidden Markov model with multiple observations). It uses a population code for encoding the log-posterior estimation of the time varying hidden stimulus, which is modeled as a continuous RV instead of the binary hidden state used in [17]. In these studies, as in ours, spikes times carry relevant information, although there the spikes are generated deterministically and signal a prediction error used to update and correct the estimated log-posterior, whereas in our approach the spikes are generated by a stochastic neuron model and define the current values of the RVs during the sampling.

The idea that nonlinear dendritic mechanisms could account for the nonlinear processing that is required in neural models that perform probabilistic inference has been proposed previously in [39] and [41], albeit for the belief propagation algorithm. In [39] the authors introduce a neural model that implements probabilistic inference in hidden Markov models via the belief propagation algorithm, and suggest that the nonlinear functions that arise in the model can be mapped to the nonlinear dendritic filtering. In [41] another rate-based neural model that implements the loopy belief propagation algorithm in general graphical models was described, where the required multiplication operations in the algorithm were proposed to be implemented by the nonlinear processing in individual dendritic trees.

While there exist several different spiking neural network models in the literature that perform probabilistic inference based on the belief propagation algorithm, there is a lack of spiking neural network models that implement probabilistic inference through Markov chain Monte Carlo (MCMC sampling). To the best of our knowledge, the neural implementations proposed in this article are the only spiking neural networks for probabilistic inference via MCMC in general graphical models. In [35] a non-spiking neural network composed of stochastic binary neurons was introduced called Boltzmann machine, that performs probabilistic inference via Gibbs sampling. The neural network in [35] performs inference via sampling in probability distributions that have only pairwise couplings between the RVs. An extension was proposed in [51], that can perform Gibbs sampling in probability distributions with higher order dependencies between the variables, which corresponds to the class of probability distributions that we consider in this article. A spiking neural network model based on the results in [35] had been proposed in [52], for a restricted class of probability distributions that only have second order factors, and which satisfy some additional constraints on the conditional independencies between the variables. To the best of our knowledge, this approach had not been extended to more general probability distributions.

A recent study [53] showed that as the noise in the neurons increases and their reliability drops, the optimal couplings between the neurons that maximize the information that the network conveys about the inputs become larger in magnitude, creating a redundant code that reduces the impact of noise. Effectively, the network learns the input distribution in its couplings, and uses this knowledge to compensate for errors due to the unreliable neurons. These findings are consistent with our models, and although we did not consider learning in this article, we expect that the introduction of learning mechanisms that optimize a mutual information measure in our neural implementations would yield optimal couplings that obey the same principles as the ones reported in [53]. While stochasticity in the neurons represents a crucial property that neural implementations of probabilistic inference through sampling rely on, this study elucidates an important additional effect it has in learning paradigms that use optimality principles like information maximization: it induces redundant representation of information in a population of neurons.

The existing gap between abstract computational models of information processing in the brain that use MCMC algorithms for probabilistic inference on one hand, and neuroscientific data about neural structures and neural processes on the other hand, has been pointed out and emphasized by several studies [12], [13], [54], [55]. The results in [1] and in this article propose neural circuit models that aim to bridge this gap, and thereby suggest new means for analyzing data from spike recordings in experimental neuroscience, and for evaluating the more abstract computational models in light of these data. For instance, perceptual multistability in ambiguous visual stimuli and several of its related phenomena were explained through abstract computational models that employ sequential sampling with the Metropolis MCMC algorithm [55]. In our simulations (see Fig. 10) we showed that a spiking neural network can exhibit multistability, where the state changes from one mode of the posterior distribution to another, even though the Markov chain defined by the neural network does not satisfy the detailed balance property (i.e. it is not a reversible Markov chain) like the Metropolis algorithm.

### Experimentally Testable Predictions of our Models

Our models postulate that knowledge is encoded in the brain in the form of probability distributions 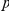, that are not required to be of the restricted form of 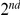 order Boltzmann distributions (5). Furthermore they postulate that these distributions are encoded through synaptic weights and neuronal excitabilities, and possibly also through the strength of dendritic branches. Finally, our approach postulates that these learnt and stored probability distributions 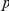 are activated through the inherent stochastic dynamics of networks of spiking neurons, using nonlinear features of network motifs and neurons to represent higher order dependencies between RVs. It also predicts that (in contrast to the model of [1]) synaptic connections between neurons are in general not symmetric, because this enables the network to encode higher order factors of 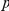.

The postulate that knowledge is stored in the brain in the form of probability distributions, sampled from by the stochastic dynamics of neural circuits, is consistent with the ubiquitous trial-to-trial variability found in experimental data [56], [57]. It has been partially confirmed through more detailed analyses, which show that spontaneous brain activity shows many characteristic features of brain responses to natural external stimuli ([3], [58], [59]). Further analysis of spontaneous activity is needed in order to verify this prediction. Beyond this prediction regarding spontaneous activity, our approach proposes that fluctuating neuronal responses to external stimuli (or internal goals) represent samples from a conditional marginal distribution, that results from entering evidence 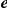 for a subset of RVs of the stored distribution 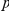 (see (1)). A verification of this prediction requires an analysis of the distributions of network responses –rather than just averaging –for repeated presentations of the same sensory stimulus or task. Similar analyses of human responses to repeated questions have already been carried out in cognitive science [60]–[62], and have been interpreted as evidence that humans respond to queries by sampling from internally stored probability distributions.

Our resulting model for neural emulations of probabilistic inference predicts, that even strong firing of a single neuron (provided it represents a RV whose value has a strong impact on many other RVs) may drastically change the activity pattern of many other neurons (see the change of network activity after 3 s in Fig. 8, which results from a change in value of the RV that represents “x-ray”). One experimental result of this type had been reported in [63]. Fig. 8 also suggests that different neurons may have drastically different firing rates, where a few neurons fire a lot, and many others fire rarely. This is a consequence both of different marginal probabilities for different RVs, but also of the quite different computational role and dynamics of neurons that represent RVs (“principal neurons”), and auxiliary neurons that support the realization of the NCC, and which are only activated by a very specific activation patterns of other presynaptic neurons. Such strong differences in the firing activity of neurons has already been found in some experimental studies, see [64], [65]. In addition, Fig. 10 predicts that recordings from multiple neurons can typically be partitioned into time intervals, where a different firing pattern dominates during each time interval, see [28], [29] for some related experimental data.

Apart from these more detailed predictions, a central prediction of our model is, that a subset of cortical neurons (the “principal neurons”) represent through their firing activity the current value of different salient RVs. This could be tested, for example, through simultaneous recordings from large numbers of neurons during experiments, where the values of several RVs that are relevant for the subject, and that could potentially be stored in the cortical area from which one records, are changed in a systematic manner.

It might potentially be more difficult to test, which of the concrete implementations of computational preprocessing for satisfying the NCC that we have proposed, are implemented in some neural tissue. Both the underlying theoretical framework and our computer simulations (see Fig. 8) predict that the auxiliary neurons involved in these local computations are rarely active. More specifically, the model predicts that they only become active when some specific set of presynaptic neurons (whose firing state represents the current value of the RVs in 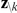) assumes a specific pattern of firing and non-firing. Implementation 3 and 5 make corresponding predictions for the activity of different dendritic branches of pyramidal neurons, that could potentially be tested through 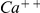-imaging.

### Conclusion

We have proposed a new modelling framework for brain computations, based on probabilistic inference through sampling. We have shown through computer simulations, that stochastic networks of spiking neurons can carry out demanding computational tasks within this modelling framework. This framework predicts specific functional roles for nonlinear computations in network motifs and dendritic computation: they support representation of higher order dependencies between salient random variables. On the micro level this framework proposes that local computational operations of neurons superficially resemble logical operations like AND and OR, but that these atomic computational operations are embedded into a stochastic network dynamics. Our framework proposes that the functional role of this stochastic network dynamics can be understood from the perspective of probabilistic inference through sampling from complex learnt probability distributions, that represent the knowledge base of the brain.

## Methods

### Markov Chains

A Markov chain 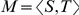 in discrete time is defined by a set 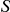 of states 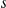 (we consider for discrete time only the case where 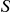 has a finite size, denoted by 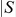) together with a transition operator 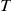. 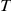 is a conditional probability distribution 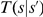 for the next state 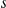 of 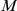, given its preceding state 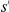. The Markov chain 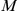 is started in some initial state 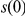, and moves through a trajectory of states 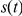 via iterated application of the stochastic transition operator 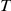 (more precisely, if 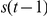 is the state at time 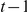, then the next state 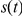 is drawn from the conditional probability distribution 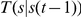. A powerful theorem from probability theory (see e.g. p. 232 in [5]) states that if 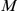 is irreducible (i.e., any state in 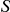 can be reached from any other state in 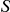 in finitely many steps with probability 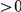) and aperiodic (i.e., its state transitions cannot be trapped in deterministic cycles), then the probability 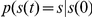 was the initial state) converges for 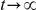 to a probability 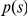 that does not depend on 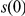. This state distribution 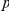 is called the stationary distribution of 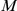. The irreducibility of 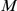 implies that 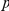 is the only distribution over the states 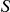 that is invariant under the transition operator 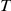, i.e.
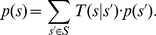
(15)Thus, in order to generate samples from a given distribution 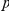, it suffices to construct an irreducible and aperiodic Markov chain 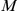 that leaves 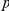 invariant, i.e., satisfies (15). This Markov chain can then be used to carry out probabilistic inference of posterior distributions of 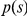 given an evidence for some of the variables in the state 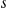. Analogous results hold for Markov chains in continuous time [5], on which we will focus in this article.

### Neuron Models

We use two types of neurons, a stochastic point neuron model as in [1], and a multi-compartment neuron model.

#### Point neuron model

We use the same point neuron model as in [1], i.e. stochastic neurons that are formalized in terms of the spike response model [16]. In [1] rigorous proofs of the validity of neural sampling were only given for spiking neurons with an absolute refractory period of length 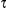 (the length of a PSP). The same holds for our results. But it was already shown in [1] that practically also a variation of the neurons model with a relative refractory period can be used. In this variation of the model one can have a quite arbitrary refractory mechanism modeled with a refractory function 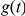, that represents the readiness of the neuron to fire within the refractory period. The firing probability of the neuron model is then
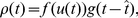
(16)where 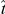 is the time of the last firing of the neuron before time 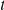. The 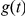 function usually has value 0 for 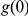, meaning that the neuron cannot fire a second spike immediately after it has fired, and its value rises until 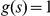 for 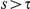, indicating that after time interval of duration 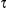 the neuron fully recovers from its refractory period (this is a slight variation of the definition of 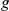 in [1]).

For a given 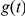 function that models the refractory mechanism, the function 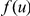 in (16) can be obtained as a solution from the equation
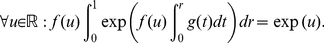
(17)It can be shown that for any continuous function 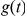 there is a unique continuous function 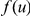 that satisfies this equation (see [1]). The multiplicative refractory function 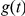 together with a modified firing probability function 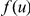 were derived in [1] to ensure that each neuron performs correct local computations and generates correct samples from the desired probability distribution if one assumes that the other neurons do not change their state. This does not guarantee in the general case that the global computation of the network when all neurons operate simultaneously generates correct samples. Nevertheless, as in [1], we observed no significant deviations from the correct posteriors in our simulations.

#### Multi-compartment neuron model

For the neural implementations with dendritic computation (Implementations 3 and 5) we used a multi-compartment neuron model which is a modified version of the neuron model introduced in [23]. It extends the stochastic point neuron model described above (with separate compartments that represent the dendritic branches) in order to capture the nonlinear effects in the integration of synaptic inputs at the dendritic branches of CA1 pyramidal neurons reported in [22] for radial oblique dendrites.

The local membrane voltage 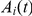 of the branch 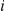 has a passive component 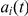 equal to the summation of the PSPs elicited by the spikes at the local synaptic inputs
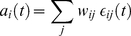
(18)where 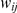 is the synaptic efficacy of input 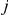 to branch 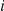 and 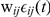 is the postsynaptic potential elicited in the branch 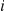 by the spikes from input 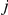. We model 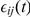 as
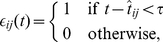
(19)where 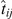 is the time of the last spike before 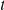 that arrived at input 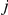. If a synchronous synaptic input from many synapses at one branch exceeds a certain threshold, the membrane voltage at the branch exhibits a sudden jump due to regenerative integration processes resulting in a dendritic spike [22]. This nonlinearity is modeled by a second active component 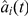


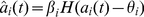
(20)where 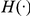 denotes the Heaviside step function, and 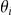 is the threshold of branch 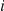. The branch potential 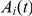 is equal to the sum of the passive component and the active component caused by the dendritic spike
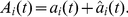
(21)


The passive and active components contribute with a different weighting factor to the membrane potential at the soma. The passive component is conducted passively with a weighting factor 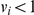 that models the attenuation of the passive signal. We assume in the neural implementations that the attenuation of the passive signal is strong, i.e. that 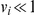. The dendritic spike is scaled by the branch strength 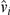. The membrane potential at the soma of the neuron is a sum of the active and passive contributions from all branches
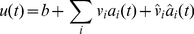
(22)The firing probability in this neuron model and its refractory mechanism are the same as for the point neuron model described above. It also can have an arbitrary refractory mechanism defined with the “eadiness to fire”multiplicative function 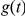 and a modified firing probability 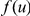.

### Details to Second Order Boltzmann Distributions with Auxiliary Variables (Implementation 1)

Let 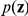 be a probability distribution
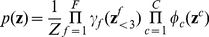
(23)that contains higher order factors, where 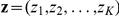 is a vector of binary RVs. 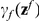 are the factors that depend on one or two RVs, and 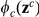 are the higher order factors that depend on more than 2 RVs. 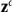 is the vector of the RVs 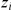 in the factor 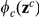, 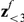 is the vector of RVs 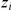 that the factor 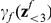 depends on, and 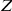 is the normalization constant. 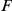 is the number of first and second order factors, and 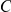 is the total number of factors of order 3 or higher. To simplify the notation, in the following we set 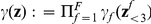, since this set of factors in 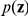 will not be changed in the extended probability distribution.

Auxiliary RVs are introduced for each of the higher order factors. Specifically, the higher order relation of factor 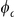 is represented by a set of auxiliary binary RVs 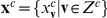, where we have a RV 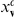 for each possible assignment 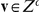 to the RVs in 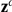 (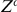 is the domain of values of the vector 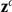). With the additional sets of RVs 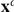 we define a probability distribution 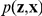 as
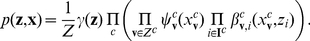
(24)We denote the ordered set of indices of the RVs that compose the vector 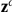 as 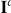, i.e.
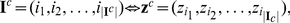
(25)where 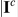 denotes the number of indices in 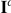.

The second order factors 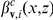 are defined as
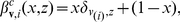
(26)where 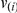 denotes the component of the assignment 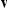 to 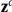 that corresponds to the variable 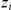, and 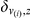 is the Kronecker-delta function. The factors 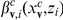 represent a constraint that if the auxiliary RV 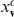 has value 1, then the values of the RVs in the corresponding factor 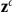 must be equal to the assignment 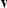 that 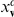 corresponds to. If all components of 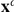 are zero, then there is not any constraint on the 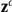 variables. This implies another property: at most one of the RVs 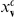 in the vector 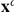, the one that corresponds to the state of 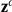, can have value 1. Hence, the vector 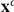 can have two different states. Either all its RVs are zero, or exactly one component 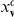 is equal to 1, in which case one has 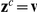. The probability 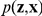 for values of 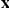 and 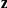 that do not satisfy these constraints is 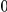.

The values of the factors 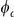 in 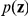 for various assignments of 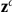 are represented in 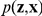 by first order factors that depend on a single one of the RVs 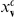. For each 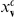 we have a new factor with value 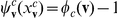 if 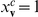, and 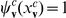 otherwise. We assume that the original factors are first rescaled, such that 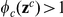 for all values of 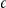 and 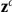. We had to modify the values of the new factors by subtracting 1 from the original value 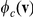, because we introduced an additional zero state for 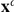 that is consistent with any of the possible assignments of 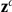.

The resulting probability distribution 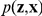 consists of first and second order factors.

#### Proposition

*The distribution* 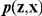 *defined in* (24) *has* 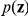 *as a marginal distribution, i.e. satisfies* (9).

*Proof.* If 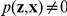, then for each 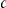 either 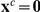 (where 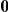 denotes the zero vector), or 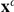 has one component 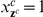, and 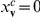 for all 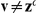. The latter value of 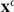 we denote as 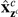. For all other values of 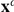 we have 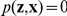. Hence
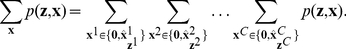
(27)Further, if we substitute the definition of the factors 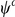 in (24), for pairs of vectors 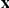 and 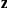 such that 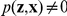 (i.e. when 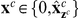 for all 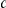) we have
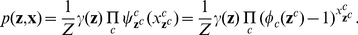
(28)Hence we can rewrite (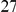) as
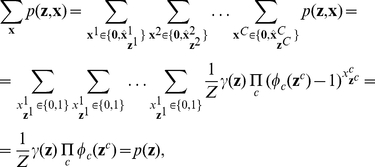
(29)yielding a proof of (9).

The resulting spiking neural network 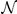 consists of principal neurons 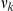, one for each of the original RVs 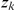, and one principal neuron 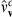 for each of the auxiliary RVs 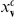. If we assume that the factor 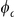 depends on 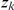, then the deterministic constraint that governs the relation between 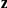 and 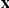 is implemented by very strong excitatory connections 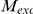 (ideally equal to 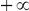) between the principal neuron 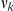 and all principal neurons 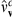 for which 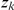 is 1 in the assignment 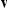 to 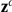. If for the principal neuron 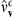 in the corresponding assignment 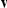 to 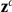 the value of 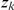 is 0, then there are strong inhibitory connections 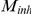 (ideally equal to 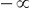) through an inhibitory interneuron between neuron 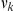 and neuron 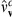. Additionally, each of the principal neurons 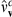 has a bias
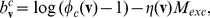
(30)where the function 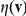 denotes the number of coordinates of the vector 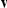 that have value 1. The biases of the principal neurons 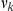 and the efficacies of the direct synaptic connections between the principal neurons 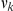 that correspond to the second order factors in 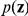 are determined in the same way as for the spiking neural network structure in [1] and depend only on the first and second order factors of 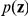.

#### Proposition

*The Markov chain represented by the spiking neural network that performs neural sampling in the Boltzmann distribution* 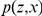 *is irreducible.*

*Proof.* We designate a state of the neural network with the vector 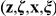. Here 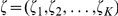, where 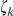 is the refractory variable of the principal neuron 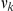, and 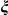 is a vector of all refractory variables 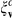 for the principal neurons 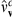 that correspond to the auxiliary RVs 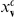. The latter are defined as in [1]. At each spike of a neuron its refractory variable is set to 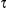 (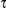 in neural sampling in discrete time is an integer number, that denotes the duration of the PSP in terms of discrete time steps). It decreases by 1 at each subsequent time step, until it reaches 0. We denote the transition operators for the refractory variables 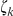 changing from state 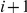 to 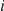 with 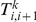, and changing from state 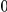 to 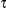 with 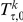. For the refractory variables 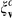 the transition operators are 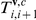 and 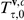. In the proof we consider the ideal case where 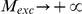 and 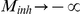, which can result in infinitely large membrane potentials equal to 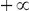 or 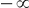. These values of the membrane potentials forbid the neuron to change the value of its RV, because if 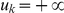 then 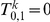, and if 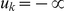 then 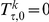 (see [1] for details), and the neuron is locked to one value of the RV. In all other cases, when the value of the membrane potential remains finite, we have 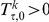 and 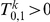. In this case the principal neuron can reach any value of 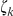 from any other value in at most 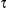 time steps. The same holds for the principal neurons 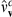.

If we consider now an initial arbitrary non-forbidden state 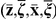, then each refractory variable 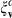 with 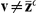 is equal to 0, and 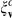 with 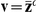 can be either non-zero or 0. If 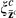 is non-zero then, since the membrane potential of the principal neuron 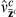 is 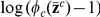, which is finite, there is a non-vanishing probability for the network state 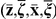 to change to another state in which 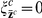 in at most 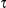 time steps. Therefore we can conclude, that from the state 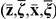 we can reach the state 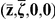 that has 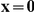 and 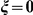 in at most 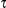 time steps with a non-vanishing probability. In this new state all principal neurons 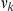 are allowed to change the value of their RV, because their membrane potentials have finite values determined by the sum of their biases and the efficacies of the synaptic connections from the second order factors. Hence each non-zero 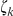 can change its value to 0 in at most 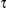 time steps. From this it follows that from any non-forbidden state 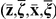 we can reach the zero state 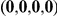 in at most 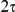 time steps with non-vanishing probability.

We proceed in a similar manner to prove that from the zero state we can reach any other non-forbidden state 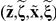. First we observe that from the zero state the principal neurons 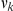 can change their states 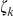 to 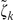 in at most 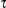 time steps, since they all have finite membrane potentials, i.e. we can reach the state 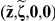. From the state 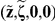 there is non-vanishing probability that the Markov chain goes in the next 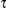 time steps through a sequence of subsequent states that all have 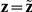. If the Markov chain follows such a sequence, then the state after exactly 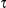 time steps has also 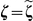. Additionally, if the Markov chain goes through such a sequence of states, at each of the 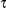 time steps after the state 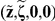 the principal neurons 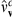 with 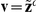 will have finite membrane potentials equal to 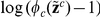. Therefore, there is non-vanishing probability that they change their states 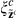 to 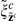 in exactly 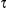 steps. Hence, we have shown that we can reach the state 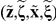 from the state 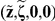 in exactly 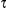 number of states. This concludes the proof that we can reach any non-forbidden state 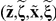 from any other other non-forbidden state 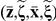 in at most 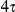 steps with non-vanishing probability, i.e. the Markov chain is irreducible.

### Details to Implementation 2

In this neural implementation each principal neuron 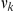 has a dedicated preprocessing layer of auxiliary neurons with lateral inhibition. All neurons in the network are stochastic point neuron models.

The auxiliary neurons for the principal neuron 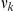 receive as inputs the outputs of the principal neurons corresponding to all RVs in the Markov blanket of 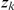. The number of auxiliary excitatory neurons that connect to the principal neuron 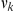 is 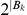 (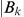 is the number of elements of 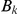), and we index these neurons with all possible assignments of values to the RVs in the vector 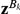. Thus, for each state 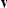 of values at the inputs 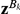 we have a corresponding auxiliary neuron 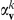. The realization of the NCC is achieved by a specific connectivity between the inputs and the auxiliary neurons and appropriate values for the intrinsic excitabilities of the auxiliary neurons, such that at each moment in time only the auxiliary neuron 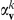 corresponding to the current state of the inputs 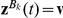, if it is not inhibited by the lateral inhibition due to a recent spike from another auxiliary neuron, fires with a probability density as demanded by the NCC (3):
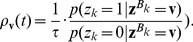
(31)During the time when the state 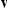 of the inputs is active, the other auxiliary neurons are either strongly inhibited, or do not receive enough excitatory input to reach a significant firing probability.

The inputs connect to the auxiliary neuron 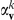 either with a direct strong excitatory connection, or through an inhibitory interneuron 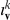 that connects to the auxiliary neuron. The inhibitory interneuron 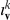 fires whenever any of the principal neurons of the RVs 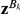 that connect to it fires. The auxiliary neuron 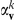 receives synaptic connections according to the following rule: if the assignment 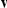 assigns a value of 1 to the RV 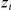 in the Markov blanket 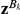, then the principal neuron 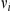 connects to the neuron with a strong excitatory synaptic efficacy 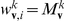, whereas if 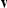 assigns a value of 0 to 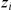 then the principal neuron 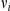 connects to the inhibitory interneuron 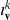. Thus, whenever 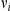 fires, the inhibitory interneuron fires and prevents the auxiliary neuron 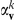 to fire for a time period 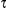. We will assume that the synaptic efficacy 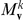 is much larger than the log-odd ratio value of the RV 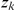 given 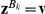 according to the r.h.s. of (3). We set the bias of the auxiliary neuron 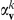 equal to
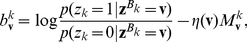
(32)where 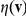 gives the number of components of the vector 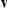 that are 1.

If the value of the inputs at time 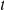 is 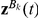, and none of the neurons fired in the time interval 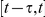, then for an auxiliary neuron 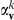 such that 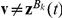 there are two possibilities. Either there exists a component of 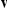 that is 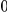 and its corresponding input 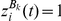, in which case the principal neuron of the RV 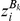 connects to the inhibitory interneuron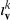 and inhibits 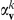. Or one has 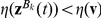 in which case the number of active inputs that connect to neuron 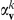 do not provide enough excitatory input to reach the high threshold for firing. In this case the firing probability of the neuron 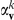 is
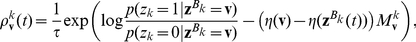
(33)and because of the strong synaptic efficacies of the excitatory connections equal to 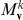, which are by definition much larger than the log-odd ratio of the RV 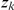, it is approximately equal to 0. Hence, only the neuron 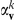 with 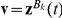 has a non-vanishing firing probability equal to (31).

The lateral inhibition between the auxiliary neurons is implemented through a common inhibitory circuit to which they all connect. The role of the lateral inhibition is to enforce the necessary refractory period of 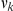 after any of the auxiliary neurons fires. When an auxiliary neuron fires, the inhibitory circuit is active during the duration of the EPSP (equal to 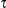), and strongly inhibits the other neurons, preventing them from firing. The auxiliary neurons connect to the principal neuron 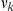 with an excitatory connection strong enough to drive it to fire a spike whenever any one of them fires. During the time when the state of the input variables satisfies 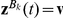, the firing probability of the auxiliary neuron 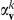 satisfies the NCC (3). This implies that the principal neuron 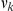 satisfies the NCC as well.

Introducing an evidence of a known value of a RV in this model is achieved by driving the principal neuron with an external excitatory input to fire a spike train with a high firing rate when the observed value of the RV is 1, or by inhibiting the principal neuron with an external inhibitory input so that it remains silent when the observed value of the RV is 0.

### Details to Implementation 3

We assume that the principal neuron 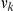 has a separate dendritic branch 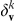 for each possible assignment of values to the RVs 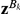, and that the principal neurons corresponding to the RVs 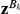 in the Markov blanket 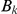 connect to these dendritic branches.

It is well known that synchronous activation of several synapses at one branch, if it exceeds a certain threshold, causes the membrane voltage at the branch to exhibit a sudden jump resulting from a dendritic spike. Furthermore the amplitude of such dendritic spike is subject to plasticity [22]. We use a neuron model according to [23], that is based on these experimental data. The details of this multi-compartment neuron model were presented in the preceding subsection of Methods on Neuron Models. We assume in this model that the contribution of each dendritic branch to the soma membrane voltage is predominantly due to dendritic spikes, and that the passive conductance to the soma can be neglected. Thus, according to (22), the membrane potential at the soma is equal to the sum of the nonlinear active components contributed from each of the branches 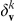:
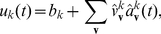
(34)where 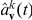 is the nonlinear contribution from branch 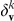, and 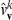 is the strength of branch 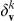 (see [22] for experimental data on branch strengths). 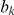 is the target value of the membrane potential in the absence of any synaptic input. The nonlinear active component (dendritic spike) 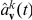 is assumed to be equal to
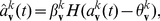
(35)where 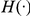 denotes the Heaviside step function, 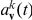 is the local activation, and 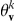 is the threshold of branch 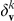. The amplitude of the total contribution of branch 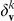 to the membrane potential at the soma is then 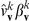.

As can be seen in Fig. 4, the connectivity from the inputs to the dendritic branches is analogous as in Implementation 2 with auxiliary neurons: from each principal neuron 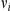 such that 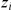 is in the Markov blanket of 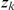 there is a direct synaptic connection to the dendritic branch 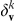 if the assignment 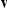 assigns to 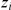 the value 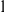, or a connection to the inhibitory interneuron 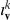 in case 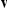 assigns the value 0 to 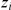. The inhibitory interneuron 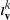 connects to its corresponding branch 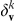, and fires whenever any of the principal neurons that connect to it fire. The synaptic efficacies of the direct synaptic connections are assumed to satisfy the condition
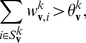
(36)where 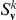 is the set of indices of principal neurons 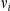 that directly connect to the dendritic branch 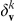, 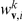 is the efficacy of the synaptic connection to the branch from 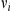, and 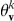 is the threshold at the dendritic branch for triggering a dendritic spike. Additionally, each synaptic weight 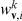 should also satisfy the condition
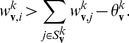
(37)The same condition applies also for the efficacy 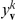 of the synaptic connection from inhibitory interneuron 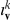 to the dendritic branch 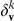.

These conditions ensure that if the current state of the inputs is 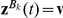, then the dendritic branch 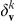 will have an active dendritic spike, whereas all other dendritic branches will not receive enough total synaptic input to trigger a dendritic spike. The amplitude of the dendritic spike from branch 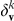 at the soma is
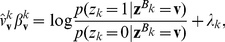
(38)where 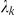 is a positive constant that is larger than all possible negative values of the log-odd ratio. If the steady value of the membrane potential is equal to 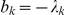, then we have at each moment a membrane potential that is equal to the sum of the amplitude of the nonlinear contribution of the single active dendritic branch and the steady value of the membrane potential, which yields the expression for the NCC (4).

### Details to the Implementation 4

In this implementation a principal neuron 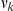 has a separate group of auxiliary neurons for each factor 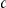 that depends on the variable 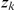. The group of auxiliary neurons for the factor 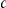 receives inputs from the principal neurons that correspond to the set of the RVs 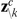 that factor 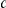 depends on, but without 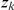. For each possible assignment of values 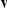 to the inputs 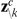, there is an auxiliary neuron in the group for the factor 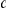, which we will denote with 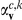. The neuron 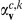 spikes immediately when the state of the inputs switches to 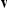 from another state, i.e. the spike marks the moment of the state change. This can be achieved by setting the bias of the neuron similarly as in (32) to 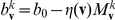 where 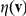 is the number of components of the vector 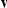 that are equal to 1, 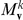 is the efficacy of the direct synaptic connections from the principal neurons to 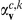 and 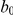 is a constant that ensures high firing probability of this neuron when the current value of the inputs is 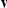.

The connectivity from the auxiliary neurons to the principal neuron keeps the soma membrane voltage of the principal neuron 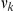 equal to the log-odd ratio of 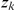 ( = r.h.s. of (4)). From each auxiliary neuron 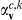 there is one excitatory connection to the principal neuron, terminating at a separate dendritic branch 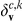. The efficacy of this synaptic connection is 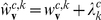, where 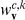 is the parameter from (13), and 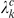 is a constant that shifts all these synaptic efficacies 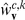 into the positive range.

Additionally, there is an inhibitory interneuron 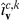 connecting to the same dendritic branch 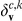. The inhibitory interneuron 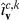 receives input from all other auxiliary neurons in the same sub-circuit as the auxiliary neuron 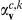, but not from 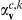. The purpose of this inhibitory neuron is to shunt the active EPSP when the inputs 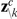 change their state from 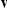 to another state 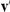. Namely, at the time moment when the inputs change to state 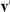, the corresponding auxiliary neuron 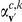 will fire, and this will cause firing of the inhibitory interneuron 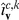. A spike of the inhibitory interneuron should have just a local effect: to shunt the active EPSP caused by the previous state 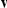 at the dendritic branch 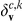. If there is not any active EPSP, this spike of the inhibitory interneuron should not affect the membrane potential at the soma of the principal neuron 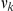.

At any time 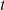, the group of auxiliary neurons for the factor 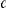 contributes one EPSP to the principal neuron, through the synaptic input originating from the auxiliary neuron that corresponds to the current state of the inputs 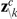. The amplitude of the EPSP from the sub-circuit that corresponds to the factor 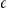 is equal to 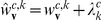. If we assume that the bias of the soma membrane potential is 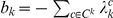, then the total membrane potential at the soma of the principal neuron 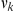 is equal to:
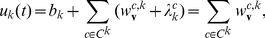
(39)which is equal to the expression on the r.h.s. of (13) when one assumes that 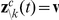. Hence, the principal neuron 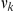 satisfies the NCC.

### Details to the Implementation 5

In this implementation each principal neuron is a multi-compartment neuron of the same type as in Implementation 3, with a separate group of dendritic branches for each factor 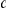 in the probability distribution that depends on 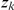. In the group 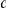 (corresponding to factor 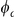) there is a dendritic branch 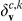 for each assignment 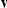 to the variables 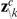 that the factor 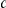 depends on (without 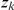). The dendritic branches in group 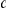 receive synaptic inputs from the principal neurons that correspond to the RVs 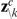. Each dendritic branch 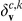 can contribute a component 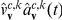 to the soma membrane voltage 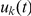 (where 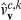 is like in Implementation 3 the branch strength of this branch), but only if the local activation 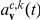 in the branch exceeds the threshold for triggering a dendritic spike. The connectivity from the principal neurons corresponding to the RVs 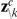 to the dendritic branches of 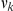 in the group 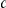 is such so that at time 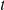 only the dendritic branch corresponding to the current state of the inputs 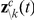 receives total synaptic input that crosses the local threshold for generating a dendritic spike and initiates a dendritic spike. This is realized with the same connectivity pattern from the inputs to the branches as in Implementation 3 depicted in Fig. 4. The amplitude of the dendritic spike of branch 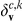 at the soma should be 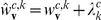 where 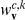 is the parameter from (13) and 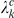 is chosen as in Implementation 3.

The membrane voltage at the soma of the principal neuron 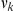 is then equal to the sum of the dendritic spikes from the active dendritic branches. At time 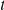 there is exactly one active branch in each group of dendritic branches, the one which corresponds to the current state of the inputs. If we additionally assume that the bias of neuron 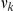 is 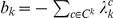, then the membrane voltage at the soma has the desired value (39).

### Details to Computer Simulations

#### Details to Computer Simulation I

The simulations with the neural network that corresponds to the approach where the firing of the principal neurons satisfies the NCC were performed with the ideal version of the implementations 2–5, which assumes using rectangular PSPs and no delays in the synaptic connections. In the simulation with the neural network that corresponds to Implementation 1, the network was also implemented with the ideal version of neural sampling. In both cases the duration of the rectangular PSPs was 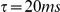 and the neurons had absolute refractory period of duration 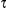. The simulations lasted for 6 seconds biological time, where in the first 3 seconds the RV for the contour (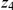) was clamped to 1 and in the second 3 seconds clamped to 0. For each spiking neural network 10 simulation trials were performed, each time with different randomly chosen initial state. The values of the synaptic efficacies 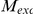 and 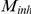 in the simulation of Implementation 1 were set to 10 times the largest value of any of the factors in the probability distribution. This ensures that a neuron with active input from a synapse with efficacy 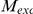 will have a very high membrane potential and will continuously stay active regardless of the state of the other inputs, and accordingly a neuron with active input from a synapse with efficacy 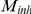 will remain silent regardless of the state of the other inputs. The time step in the simulations was set to 1 ms.

The values for the conditional probabilities 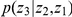 and 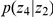 in the Bayesian network from Fig. 1 used in these simulations are given in Table 1. The prior probabilities 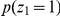 and 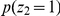 were both equal to 0.5.

**Table 1:** Values for the conditional probabilities in the Bayesian network in Fig. 1B used in Computer Simulation I.

	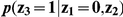	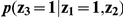	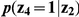
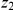 = 0	0.15	0.85	0.15
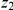 = 1	0.85	0.15	0.85

#### Details to Computer Simulation II

The conditional probability tables of the ASIA-network used in the simulations are given in Tables 2,3,4 and 5. We modified the original network from [24] by eliminating the “tuberculosis or cancer?” RV in order to get it in suitable form to be able to perform neural sampling in it. In the original ASIA network the “tuberculosis or cancer?” RV had deterministic links with the RVs “tuberculosis?” and “cancer?” which results in a Markov chain that is not connected. The model captures the following qualitative medical knowledge facts:

1. Shortness of breath or dyspnoea may be due to tuberculosis, lung cancer or bronchitis, none of them or many of them at the same time.
2. A recent visit to Asia increases the chance for tuberculosis.
3. Smoking is a risk factor for both lung cancer and bronchitis.
4. Tuberculosis and lung cancer significantly increase the chances of a positive chest x-ray test.

**Table 2:** Values for the probabilities 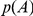, 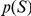 and 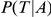 in the ASIA Bayesian network used in Computer Simulation II.

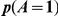	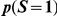	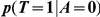	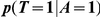
0.01	0.5	0.01	0.05

**Table 3:** Values for the conditional probabilities 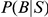 and 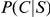 in the ASIA Bayesian network used in Computer Simulation II.

	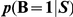	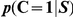
S = 0	0.3	0.01
S = 1	0.6	0.10

**Table 4:** Values for the conditional probabilities 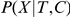 in the ASIA Bayesian network used in Computer Simulation II.

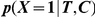	C = 0	C = 1
T = 0	0.05	0.98
T = 1	0.98	0.98

**Table 5:** Values for the conditional probabilities 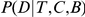 in the ASIA Bayesian network used in Computer Simulation II.

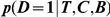	T = 0	T = 1
C = 0, B = 0	0.1	0.7
C = 0, B = 1	0.8	0.9
C = 1, B = 0	0.7	0.7
C = 1, B = 1	0.9	0.9

We used a point neuron model as in [1] described in the Introduction section of this article, where the membrane potential of the neuron is a linear sum of the PSPs elicited by the input spikes. We performed all simulations with three different shapes for the EPSPs. The first EPSP was an alpha shaped EPSP curve 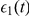 defined as
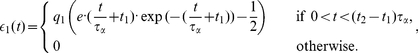
(40)where the 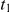 and 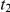 are the points in time where the alpha kernel 
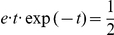
, 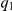 = 2.3 is a scaling factor and 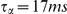 is the time constant of the alpha kernel. The second used EPSP was a plateau shaped curve 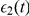 defined with the following equation
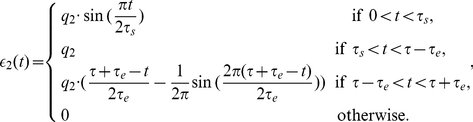
(41)where 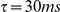. The 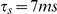 defines the duration of the rise of the EPSP kernel after an input spike, 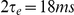 determines the duration of part of the EPSP curve corresponding to the fall of the EPSP back to the baseline, modeled here with the sine function, and 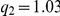 is a scaling factor. The third shape of the EPSP that we used was the theoretically optimal rectangular shape with duration 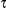. In all simulations for each of the three different shapes of EPSPs we used the same duration 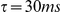 to calculate the generated samples from the spike times according to (2). All neurons had an absolute refractory period of duration 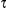. The time step in the simulations was 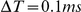.

The indirect connections going through inhibitory interneurons from the principal neurons to the auxiliary neurons were modeled as direct connections with negative synaptic efficacies and IPSPs that match the shape of the EPSPs described above. All connections in the network had delays equal to 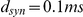. The excitatory synaptic weight from the principal neuron 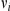 to an auxiliary neuron 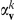 was set to
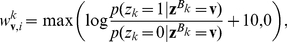
(42)and the synaptic weight for the inhibitory synaptic connection from the principal neuron 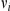 to an auxiliary neuron 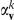 (which models the indirect inhibitory connection through the inhibitory interneuron 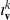) was set to
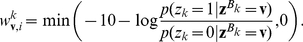
(43)


The efficacy of the synaptic connections from the auxiliary neurons to their principal neuron were set to 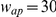. The lateral inhibition was implemented by a single inhibitory neuron that receives excitatory connections from all auxiliary neurons with synaptic efficacy equal to 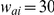. The inhibitory neuron connected back to all auxiliary neurons and these synaptic connections had rectangular shaped IPSPs with duration 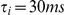. These rectangular IPSPs approximate the effect that a circuit of fast-spiking bursting inhibitory neurons with short IPSPs would have on the membrane potential of the auxiliary neurons. The efficacy of the synaptic connection from the inhibitory neuron for the lateral inhibition to the auxiliary neuron 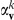 was set equal to 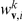 in the previous equation. The bias of the principal neurons were set to 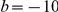 and the biases of the auxiliary neurons were set according to (32). The inhibitory interneuron for the lateral inhibition had bias 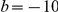.

The evidence about known RVs in the neural network was introduced by injected constant current in the corresponding principal neurons of amplitude 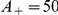 if the value of the RV is 1 and 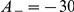 if the value of the RV is 0. The simulations were performed for 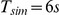 biological time. For the separate cases of each EPSP shape the results were averaged over 20 simulation trials with different initial states of the spiking neural network and different random noise throughout the simulation. The initial states were randomly chosen from the prior distribution of the ASIA network which corresponds to a random state in the activity of the spiking network when no evidence is introduced. For control we performed the same simulations with randomly chosen initial states from a uniform distribution, and the results showed slightly slower convergence (data not shown). The initial states were set by injecting constant current pulse in the principal neurons for the unknown RVs at the beginning of the simulation, with amplitude 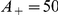 ( 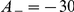 ) if the value of the RV in the initial state is 1 (0), and duration equal to 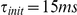.

The simulations in Computer Simulation II were performed with the PCSIM simulator for neural circuits (web site: http://www.igi.tugraz.at/pcsim) [66].

#### Details to Computer Simulation III

The simulations were performed with the ideal implementation of the NCC, which corresponds to using rectangular PSPs and zero delays in the synaptic connections in the implementations 2–5. We performed 10 simulations with an implementation that uses the neuron model with relative refractory period and another 10 simulations with an implementation that uses the neuron model with absolute refractory period. The duration of the PSPs was 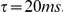 The time step of the simulation was 1 ms.

The Bayesian network in this simulation was randomly generated with a variation of the Markov chain Monte Carlo sampling algorithm proposed in [27]. Instead of allowing arcs in the Bayesian network in both directions between the nodes and checking at each new iteration whether the generated Bayesian network graph is acyclic like in [27], we preserved an ordering of the nodes in the graph and allow an edge from the node 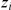 to the node 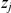 only if 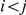. We started with a simple connected graph where each node 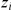, except for the first node 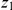, has connection from node 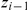. We then performed the following MCMC iterations.

1. Choose randomly a pair of nodes 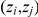 where 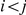;
2. If there is an edge from 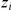 to 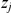 then remove the edge if the Bayesian network remains connected, otherwise keep the same Bayesian network from the previous iteration;
3. If there is not an edge, then create an edge from 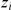 to 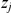 if the node 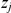 has less than 8 parents, otherwise keep the Bayesian network from the previous iteration.

Similarly to the proofs in [27], one can prove that the stationary distribution of the above Markov chain is a uniform distribution over all valid Bayesian networks that satisfy the constraint that a node can not have more than 8 parents. To generate the Bayesian network used in the simulations we performed 500000 iterations of the above Markov chain. The conditional probability distributions for the Bayesian network were sampled from Dirichlet distributions with priors 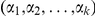 where 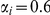 for all 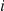.

In the simulations that use a neuron model with a relative refractory mechanism, we used the following form for the refractory function 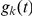


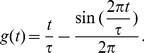
(44)The corresponding function 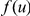 for the firing probability was calculated by numerically solving the equation (17) for 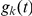 defined in (44).
